# Cultivation and metabolic versatility of novel and ubiquitous chemolithoautotrophic *Campylobacteria* from mangrove sediments

**DOI:** 10.1128/spectrum.00367-25

**Published:** 2025-07-23

**Authors:** Liang Cui, Yangsheng Zhong, Yufei Li, Stefan M. Sievert, Zhaobin Huang, Wanpeng Wang, Maxim Rubin-Blum, Xiaxing Cao, Yong Wang, Zongze Shao, Qiliang Lai, Shasha Wang, Lijing Jiang

**Affiliations:** 1 Key Laboratory of Marine Genetic Resources, Third Institute of Oceanography Ministry of Natural Resources 118477 , Xiamen, China; 2 Biology Department, Woods Hole Oceanographic Institution 10627, Woods Hole, Massachusetts, USA; 3 College of Oceanology and Food Science, Quanzhou Normal University 117823, , Quanzhou, China; 4 Biology Department, Israel Oceanographic and Limnological Research Institute 54620, Haifa, Israel; 5 Fujian Ocean Innovation Center, Xiamen, China; Guangdong Academy of Sciences, Guangzhou, China

**Keywords:** mangrove sediments, *Campylobacteria*, chemolithoautotrophic, biogeochemical cycles, *in situ*, metatranscriptomic

## Abstract

Chemolithoautotrophic members from the *Campylobacteria* class are dominant key players in sulfidic habitats, where they make up a stable portion of sulfide-oxidizing bacterial communities. Nevertheless, few isolates have so far been cultivated and studied *in situ*, and most are derived from chemosynthetic ecosystems, limiting our understanding of their physiological and metabolic features as well as ecological roles in the global marine environments. In this study, seven potentially new species were successfully isolated from mangrove sediments and further diverged into three potentially new genera within the class *Campylobacteria*. These isolates were obligate chemolithoautotrophs that could grow through hydrogen oxidation as well as sulfur oxidation, reduction, and disproportionation. Metabolic reconstructions revealed that these isolates contained diverse sulfide:quinone oxidoreductase and flavocytochrome c sulfide dehydrogenase for sulfide oxidation, distinct Sox gene cluster for sulfur oxidation, as well as group I, II, and IV hydrogenases for hydrogen consumption and production. Notably, these strains lacked the complete denitrification pathway, instead having all genes for nitrogen fixation, which might facilitate their survival in the nitrogen-limited mangrove sediments. Moreover, they also demonstrated the ability to adapt to low O_2_ conditions, such as a more efficient 2-oxoglutarate:ferredoxin oxidoreductase complex for CO_2_ fixation and diverse terminal oxidases including Cco, Cox, and Cyd. Metatranscriptomic analysis further confirmed their activity and different adaptation mechanisms in *in situ* mangrove sediments. Assessing their occurrences indicated that these lineages were globally distributed in hypoxic and anoxic environments and dominant members of marine and mangrove sediments. Overall, these results indicate that these new *Campylobacteria* members are metabolically versatile and play an underappreciated role in the biogeochemical cycling of carbon-rich mangrove sediments.

**IMPORTANCE:**

Chemolithoautotrophic *Campylobacteria* spp. are generally associated with sulfide-rich environments, where they play a key role in the cycling of carbon, nitrogen, and sulfur. Yet, only a limited number of cultured isolates are currently available. In this study, we isolated seven potentially new species belonging to three new genera from mangrove sediments, which significantly expanded our understanding of the species diversity within the class *Campylobacteria*. These isolates demonstrated diverse and unique metabolic potentials for CO_2_ fixation, sulfur oxidation, hydrogen oxidation, nitrogen metabolism, and oxygen respiration, making them well adapted to the sulfur-rich, nitrogen-limited, and low-oxygen habitats they inhabit. The frequent detection of these novel species in marine and mangrove sediments, as revealed by 16S rRNA gene sequences in public databases, indicates a potential preference for oxygen-limited environments. Overall, this study promotes our understanding of the *in situ* function and ecological role of *Campylobacteria*, especially in previously overlooked carbon-rich sediment ecosystems.

## INTRODUCTION

Mangrove sediments are one of the most productive “blue carbon” ecosystems, characterized as being rich in organic carbon and sulfur but limited in nitrogen (1, 2). Owing to the periodic exposure and inundation under tidal action, the environmental conditions vary profoundly on a spatiotemporal scale, particularly salinity, nutrient availability, and oxygen concentration, making the mangrove sediment ecosystem a unique biotope (2). These conditions contribute to the immense microbial diversity, driving complex nutrient cycling and biogeochemical processes (3). Early studies revealed that the phyla *Pseudomonadota* (classes *Alphaproteobacteria* and *Gammaproteobacteria*), *Desulfobacterota*, *Myxococcota*, and *Bacteroidota* were detected as the prevalent and dominant members in mangrove sediments (3). These taxa usually depend on reduced organic compounds as their energy and carbon sources (4). However, recent studies have shown that chemolithoautotrophic taxa are widely distributed and predominant groups in the sediments of different mangrove ecosystems. For example, *Sulfurovum*, *Sulfurimonas*, *Thermodesulfovibrio*, *Desulfobacterium*, and *Desulfococcus* were found to be abundant (relative abundance >1%) in surface sediments (0–20 cm) of Yunxiao mangrove, Fujian Province, China (5). Similarly, *Desulfococcus*, *Nitrosopumilus*, and *Sulfurimonas* were also dominant in mangrove surface sediments of 10–20 cm collected from six sites along the coastline of Beibu Gulf in Guangxi Province, China, with relative abundances of >1% (6). The dominance of these chemolithoautotrophs suggests that they may play important roles *in situ*. However, such knowledge was inferred from microbial communities via either 16S rRNA gene sequencing or metagenomics, and robust evidence of the activity, function, and contribution based on pure cultures in mangrove sediment ecosystems is currently missing.

Chemolithoautotrophic members of the class *Campylobacteria* (formerly known as *Epsilonproteobacteria*) are widely distributed in various marine and terrestrial environments (7). They are especially abundant in deep-sea hydrothermal vent systems, including plumes, diffuse fluids, chimneys, sediments, microbial mats, and animal-associated niches (7, 8). In addition to deep-sea vents, they have also been shown to dominate in other sulfidic and low-oxygen environments, such as the pelagic redoxcline, deep-sea cold seeps, gastrointestinal tracts of animals, and activated sludge (9). However, these studies are mainly based on the molecular ecological techniques, and few culturable strains from *Campylobacteria* are obtained. To date, representatives of five families have been cultivated, including *Thiovulaceae*, *Sulfurovaceae*, *Hydrogenimonadaceae*, *Nitratiruptoraceae*, and *Nautiliaceae* (10). Physiological characterizations indicated that most members of *Campylobacteria* were hydrogen- and/or sulfur-oxidizing chemolithoautotrophs, with some subgroups having distinctive physiological characteristics (11). However, most of the isolates of *Campylobacteria* are derived from deep-sea and shallow-water hydrothermal vents, sulfidic caves, cold seeps, or other chemosynthetic ecosystems (11), and there are few sources from other marine habitats. Thus, the cultivation of novel *Campylobacteria* strains from diverse habitats could shed light on the phylogenetic and metabolic diversity as well as their ecological roles and adaptation mechanisms in various marine environments.

In this study, we isolated seven new species of three potentially new genera in the family Thiovulaceae of the class *Campylobacteria* from carbon-rich mangrove sediments and further characterized the phenotypic and biochemical features of the isolates. We then investigated the metabolic potentials and *in situ* metabolizing activities of these new members by comparative genomics and metatranscriptomics and further examined their ecological distributions in the global oceans by searching against the available high-throughput sequencing database. This study provides new insights into the function and variability of *Campylobacteria* and highlights their importance for the biogeochemical cycling in carbon-rich sediment ecosystems.

## RESULTS AND DISCUSSION

### Enrichment and isolation of chemolithoautotrophic *Campylobacteria*

The sediment samples were collected from the mangrove wetland of Jiulong River tributary in Zhangzhou (117°45′E, 24°20′N), Fujian Province, China. Sediments were stored in a portable cooler at 4°C and transported back to the laboratory. The samples were immediately transferred into sealed bottles filled with MMJHS medium with CO_2_ as the carbon source, thiosulfate and hydrogen as energy sources, and oxygen as a terminal electron acceptor (12). After 2 days of incubation at 32℃, bacterial growth was observed in the enrichment cultures. The cells were subsequently isolated with the dilution-to-extinction method. Seven cultures in the serum vials showing growth at the highest dilution were designated as strains HSL1-2^T^, HSL-1656^T^, HSL-3221^T^, HSL1-6^T^, HSL3-7^T^, HSL3-2^T^, and HSL-1716^T^. Microscopic examination and 16S rRNA gene sequencing further confirmed the purity of the cultures (12).

### Phylogenetic analysis based on 16S rRNA gene and genome sequences

**Fig 1:**
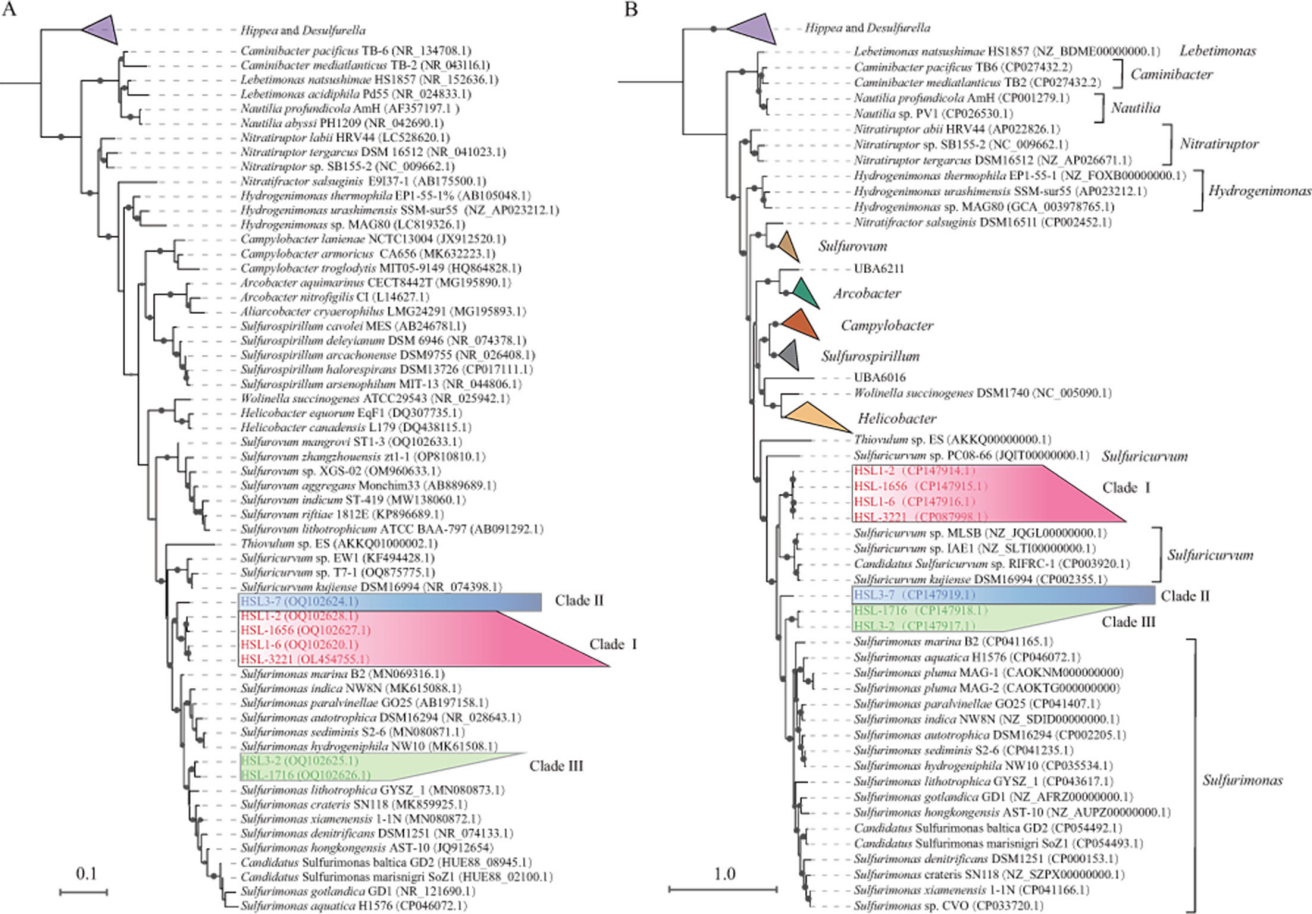
Maximum-likelihood phylogenetic tree of seven isolates based on 16S rRNA gene (**A**) and 92 core gene sequences (**B**). The text in blue, red, and green in the background represents the putative new genera, respectively. The accession numbers of the genomes are shown in parentheses. For both trees, bootstrap values of >50% are indicated as black circles at the nodes, and scale bars indicate the mean number of substitutions per site.

The 16S rRNA gene sequences of strains HSL1-2^T^, HSL-1656^T^, HSL-3221^T^, HSL1-6^T^, and HSL3-7^T^ shared the highest similarities with *Sulfurimonas marina* B2^T^ of 93.49%, 93.16%, 93.53%, 93.62%, and 93.18%, respectively. These values were below the threshold of 94.55%–95.05% for genus delineation (13), indicating that they may represent potentially new genera within the family Thiovulaceae. Furthermore, HSL3-7^T^ harbored the sequence similarities of 93.62%–94.08% with four other strains, indicating that strain HSL3-7^T^ may potentially represent a separate new genus. Strains HSL1-2^T^ (HSL-1656^T^) and HSL-3221^T^ (HSL1-6^T^) may represent two novel species within the same genus based on the threshold for species delineation of 98.65% (14). Moreover, strains HSL3-2^T^ and HSL-1716^T^ shared the highest sequence similarities with *Sulfurimonas crateris* SN118^T^ of 95.38% and 95.06%, respectively, while sharing 97.76% similarity between them, indicating that both strains may represent different new species of the genus *Sulfurimonas*. The phylogenetic tree based on the 16S rRNA gene sequences indicated that strains HSL1-2^T^, HSL-1656^T^, HSL-3221^T^, HSL1-6^T^, and HSL3-7^T^ formed a monophyletic cluster, which was distinct from the branch of the genus *Sulfurimonas* (Fig. 1A). Moreover, strain HSL3-7^T^ was clustered into a single branch, while the other four strains clustered together. Strains HSL3-2^T^ and HSL-1716^T^ were clustered with those from non-vent strains of *Sulfurimonas* (Fig. 1A). This result supported the above results of 16S rRNA gene sequence similarities.

**Fig 2:**
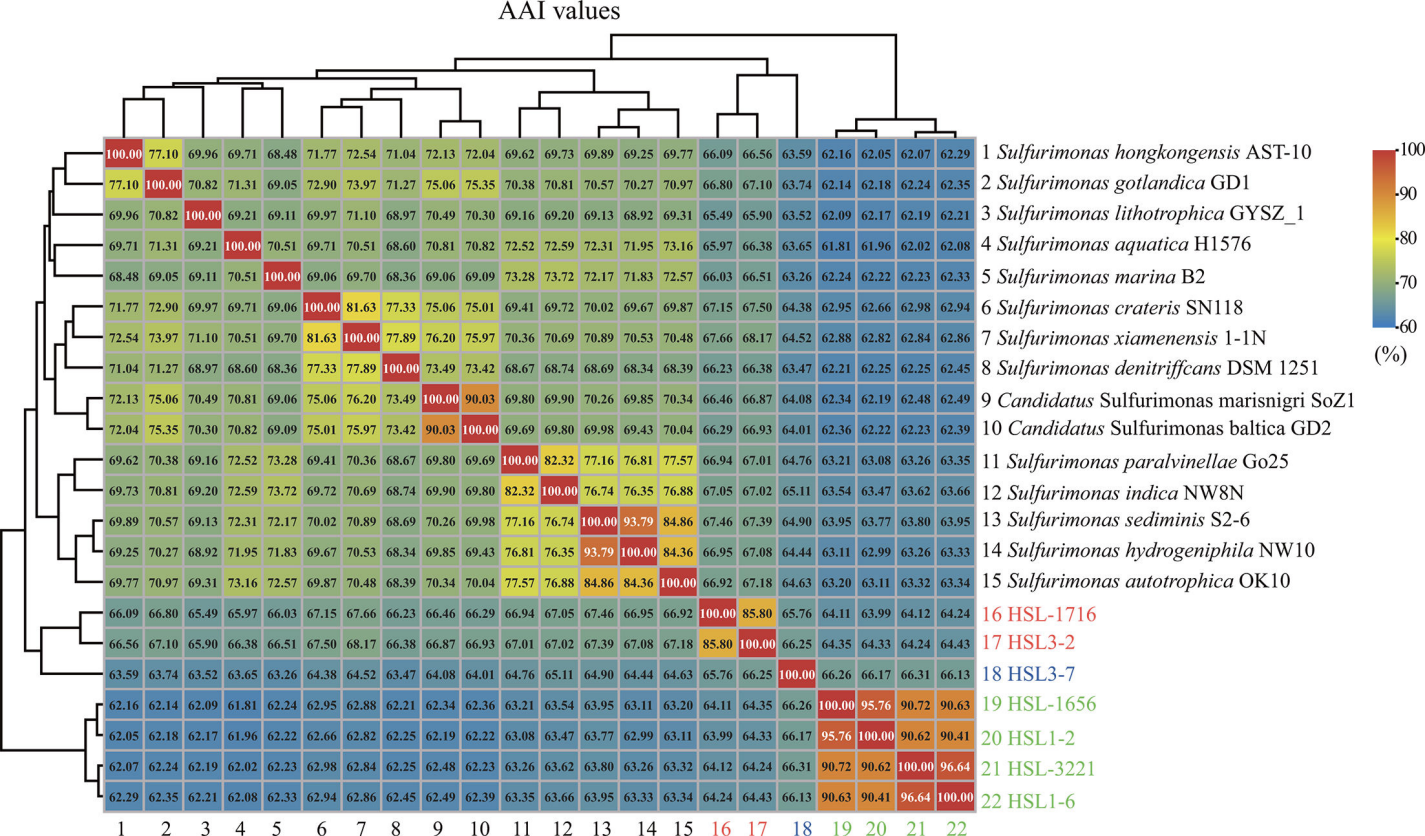
The average amino acid identity (AAI) values between genomes of seven isolated strains and the genomes of type strains of *Sulfurimonas* in the phylum Campylobacterota. The text in green, blue, and red represents the strains of clades I–III isolated in this study.

Genome-based phylogenetic analysis by up-to-date bacterial core gene (UBCG) (15) further revealed that these seven strains were well divided into three different clades within the family Thiovulaceae with high bootstrap values (Fig. 1B). Clade I (*n* = 4) comprised species-like strains HSL1-2^T^, HSL-1656^T^, HSL-3221^T^, and HSL1-6^T^, whereas clade II (*n* = 1) member was strain HSL3-7^T^. The other species, like strains HSL3-2^T^ and HSL-1716^T^, fell into clade III (*n* = 2) (Fig. 1B). Overall, three different evolutionary branches are revealed representing three potential novel genera of the class *Campylobacteria*. The taxonomic relationship of seven isolates with their related genomes was further clarified by computing digital DNA-DNA hybridization (dDDH), average nucleotide identity (ANI), and average amino acid identity (AAI) (16). Within the members of a clade, members had dDDH and ANI values of at least 13.3%–60.7% and 61.0%–94.7% (Table S2), respectively, which were below the threshold values of 70% and 95%–96% for species definition (17), suggesting that these seven strains may represent different new species within the genus. At the inter-clade level, the AAI values between seven isolated strains and the type strains from *Sulfurimonas* were in the range of 61.81%–67.46% (Fig. 2). The AAI values ranged between 66.13% and 66.31% for clades I and II, between 63.99% and 64.43% for clades I and III, and between 65.76% and 66.25% for clades II and III (Fig. 2). These values were lower than the threshold of 60%–80% for genus delineation (17), indicating that these seven strains might represent three potential novel genera. Furthermore, GTDB-Tk classification placed them in three distinct potential novel genera within the family Thiovulaceae (Table S3), which was consistent with the above results of phylogenetic analysis based on 16S rRNA gene and genome sequences.

Overall, these results indicate that these isolated strains may represent different novel species within three potential novel genera in the family *Thiovulaceae*. According to the guidelines in the International Code of Nomenclature of Prokaryotes for microbial cultivated taxa (18), we proposed the names *Thiomicrolovo*, *Sulfonitrofixus*, and *Hydrocurvibacter* for three novel genera, respectively, and *Thiomicrolovo zhangzhouensis* HSL1-2^T^, *Thiomicrolovo subterrani* HSL-1656^T^, *Thiomicrolovo sulfuroxydans* HSL-3221^T^, *Thiomicrolovo immobilis* HSL1-6^T^, *Sulfonitrofixus jiaomeiensis* HSL3-7^T^, *Hydrocurvibacter mobilis* HSL3-2^T^, and *Hydrocurvibacter sulfurireducens* HSL-1716^T^ for seven novel species, respectively.

### Phenotypic and chemotaxonomic characteristics of seven isolated strains

All seven strains were gram negative, with a coccoid to oval, rod shaped, or slightly curved morphology, between 0.4 and 1.0 μm wide and between 1.2 and 3.0 μm long (Fig. S1). The strains from *Thiomicrolovo* and *Sulfonitrofixus* were non-motile, whereas the *Hydrocurvibacter* strains were motile with a polar flagellum (Table 1), which was also reflected in the genomes as the genes encoding flagella were absent in *Thiomicrolovo* and *Sulfonitrofixus*. Furthermore, the growth temperature of all isolated strains ranged from 4℃ to 45℃ and even up to 50℃ for *T. sulfuroxydans* HSL-3221^T^, with the optimal temperature range of 30℃–37℃. The pH range for growth was 4–9, with the best growth occurring at 6–7. They could tolerate a salinity of up to 6% (wt/vol), with the best growth at 2%–3% (Table 1). There were marked differences in the utilization of electron donors and acceptors among these strains. Except for the genus *Thiomicrolovo*, all other strains from *Sulfonitrofixus* and *Hydrocurvibacter* were able to grow with H_2_ as the electron donor, with elemental sulfur, oxygen, or nitrate (only *H. sulfurireducens* HSL1716^T^) as the electron acceptors. Furthermore, all strains were able to grow with various reduced sulfur compounds such as thiosulfate, sulfite, elemental sulfur, or sulfide as the sole electron donor, with oxygen or nitrate (only *T. zhangzhouensis* HSL1-2^T^, *T. subterrani* HSL-1656^T^, and *H. sulfurireducens* HSL-1716^T^) as the sole electron acceptor. In addition, they could also disproportionate thiosulfate and elemental sulfur (Table 1), expanding the diversity of sulfur-disproportionating *Campylobacteria* beyond those previously reported from deep-sea hydrothermal vents (19). Moreover, *S. jiaomeiensis* HSL3-7^T^ could grow with H_2_ as the sole electron donor and sulfate as the electron acceptor (Fig. S2). This is the first evidence that mesophilic *Campylobacteria* can perform sulfate reduction coupling with hydrogen oxidation. Furthermore, these strains could use ammonium, nitrate, and atmospheric nitrogen as nitrogen sources but not nitrite. Heterotrophic growth showed that all seven strains did not grow in the tested organic compounds (Table 1).

**TABLE 1:** Comparison of physiological characteristics within seven isolated strains in this study.^*b*^

Characteristics	HSL1-2^T^	HSL-1656^T^	HSL-3221^T^	HSL1-6^T^	HSL3-7^T^	HSL3-2^T^	HSL-1716^T^
Shape	Coccoid to oval	Coccoid to oval	Coccoid to oval	Coccoid to oval	Rods	Rods to slightly curved	Rods to slightly curved
Motility	−	−	−	−	−	+	+
Anaerobic growth	+	+	−	−	+	+	+
Doubling time under optimal conditions (h)	4.6	1.4	3.5	2.9	3.2	12.0	10.0
Temperature range (optimal *T*) (°C)	15–45 (37)	10–40 (32)	4–50(32)	4–45 (35)	15–45 (37)	10–45 (37)	15–40 (32)
pH range (optimal pH)	5.5–8.0 (7.0)	5.0–9.0 (6.5)	4.0–9.0 (7.0)	4.5–9.0 (6.0)	4.5–8.5 (7.0)	5.4–8.6 (6.1)	5.0–9.0 (6.5–7.0)
NaCl requirement	1.0–6.0 (3.0)	2.0–4.0 (2.5)	2.0–5.0 (3.0)	1.0–4.0 (2.0)	2.0–4.0 (2.5)	1.0–5.0 (3.0)	2.0–4.0 (2.5)
Maximum O_2_ concentration (%)	20	15	15	20	10	10	15
Energy sources	S_2_O_3_^2−^, S^0^, HS^−^	S_2_O_3_^2–^, S^0^, SO_3_^2–^, HS^–^	S_2_O_3_^2–^, HS^–^	S_2_O_3_^2–^, HS^–^	H_2_, HS^–^, S_2_O_3_^2–^	H_2_, S_2_O_3_^2–^, SO_3_^2–^, S^0^	H_2_, S^0^, S_2_O_3_^2–^, HS^–^
Sulfur disproportionation	+	+	+	+	+	+	+
Organic electron donors	−	−	−	−	−	−	−
Electron acceptors	O_2_, NO_3_^–^	NO_3_^–^, O_2_	O_2_	O_2_	S_8_, O_2_, SO_4_^2–^	S_8_, O_2_	S_8_, O_2_, NO_3_^–^
GC^*a*^ content (mol%)	57.2	57.3	57.3	57.5	48.7	40.4	41.7

^*a*^ GC, guanine-cytosine.

^*b*^ +, can be used; −, cannot be used.

The cellular fatty acid composition was determined for the seven species in parallel with reference type strains, and the results are detailed in Table S4. The principal fatty acids (>10%) for all seven strains were summed feature 3 (C_16:1_ *ω*7*c*/C_16:1_ *ω*6*c*), C_16:0_ , and summed feature 8 (C_18:1_ *ω*7*c*/C_18:1_ *ω*6*c*), similar to the members from the genus *Sulfurimonas* (20). Although the fatty acid proﬁles from these strains were similar, their proportions were diﬀerent from each other (Table S4). For example, C_12:0_ comprised 1.29% of fatty acids in *S. marina* B2^T^ but was only present in trace amounts in *Thiomicrolovo* and *Sulfonitrofixus* (0.49%–0.63%). C_14:0_ 3-OH was found at high levels of 6.23%–7.14% in *Hydrocurvibacter* but not detected in *S. crateris* SN118^T^. Among these three genera, C_14:0_ was the primary fatty acid (>10%) in *Thiomicrolovo* and *Sulfonitrofixus* but only present at lower levels of 5.73%–7.29% in *Hydrocurvibacter*. C_18:0_ was detected at high levels of 5.31% and 6.19% in *Hydrocurvibacter* but only in trace amounts of 0.23%–1.58% in *Thiomicrolovo* and *Sulfonitrofixus* (Table S4).

### Genomic properties of seven isolated strains

The general genome properties of the seven newly sequenced strains are summarized in Table S5. Complete genome sequences of seven strains were obtained with a circular chromosome, and no plasmids were detected. The genome size within clade I (*n* = 4) was 2.30–2.56 Mbp; that within clade II (*n* = 1) was 2.56 Mbp; and that within clade III (*n* = 2) was 2.10–2.26 Mbp. These values were within the range of 1.92–2.95 Mbp for the genus *Sulfurimonas*, with an average genome size of 2.31 Mbp (20). The genome completeness and contamination within clade I (*n* = 4) were 99.39%–100.0% and 1.83%–2.24%; those within clade II (*n* = 1) were 99.18% and 5.28%; and those within clade III (*n* = 2) were 99.18%–99.59% and 1.22%–1.42% (Table S3). Moreover, the guanine-cytosine (GC) contents of the genomes were 57.25–58.46 mol% (clade I), 49.73 mol% (clade II), and 40.42–41.74 mol% (clade III), respectively, beyond the range of 33.2–38.8 mol% as previously reported for the members of *Sulfurimonas* (20). A previous study has proposed that genomes with a higher genomic GC content represent an adaptation to elevated high temperatures (21). Moreover, other studies have also indicated that nitrogen-fixing aerobic bacteria possess higher genomic GC content than non-fixing species within the same genus (22). A recent study indicated that the higher GC content may be attributed to the isolation caused by the density barrier or the unique physicochemical conditions (e.g., extremely high salinity) in the Kebrit Deep (23). The need to repair DNA damage induced by high salinity could lead to frequent GC-biased gene conversion, thereby resulting in a higher GC content (23). However, the specific mechanism of higher GC content in these isolated strains from mangrove sediments needs further exploration.

### Comparative genomic analyses reveal versatile metabolic potentials for isolated strains of *Campylobacteria*

#### Carbon fixation

**Fig 3:**
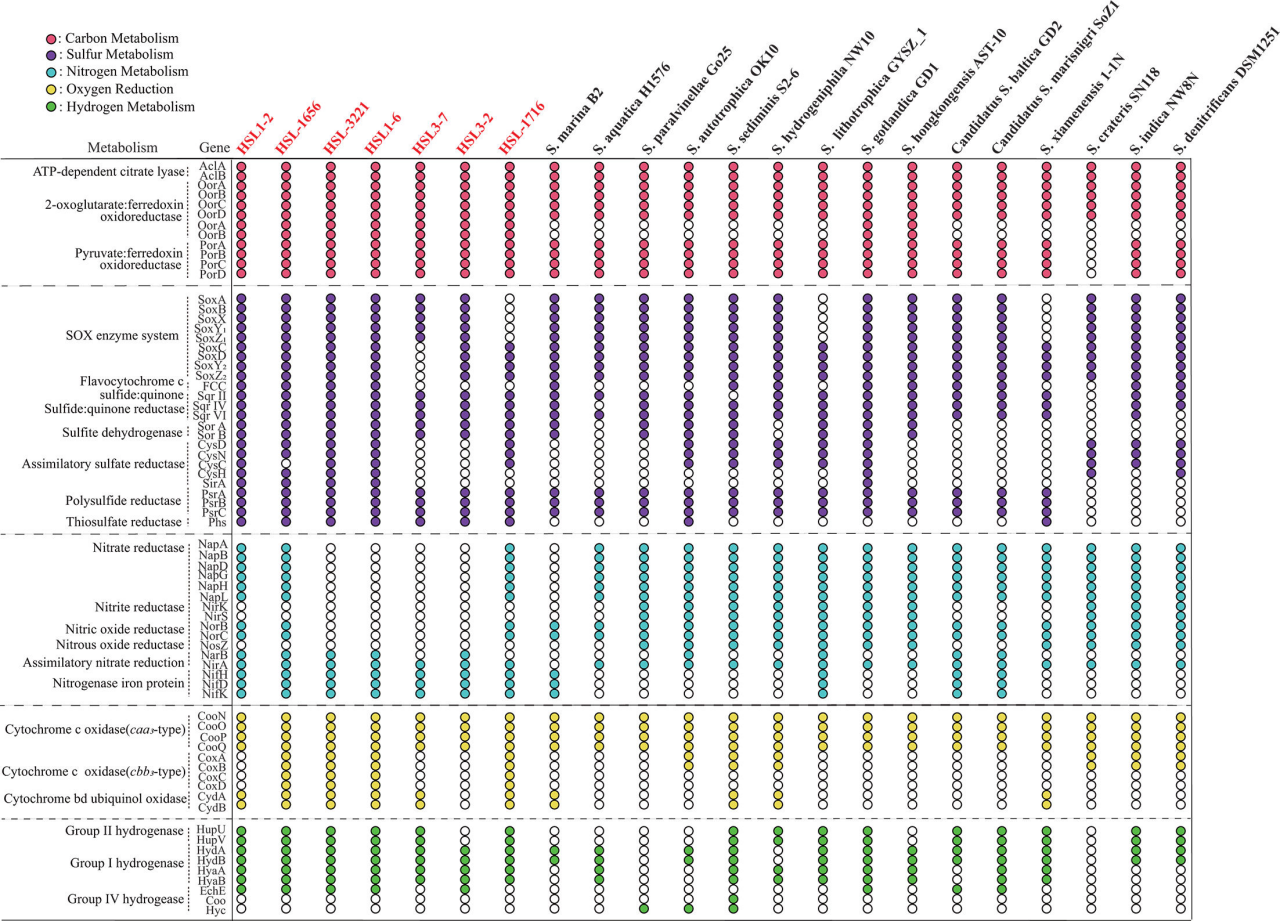
Presence of key genes in carbon fixation, sulfur metabolism, nitrogen metabolism, oxygen reduction, and hydrogen metabolism in seven strains of this study and the type strains of the genus *Sulfurimonas*. The text in red and black represents our strains and the members from *Sulfurimonas*, respectively. Circles of different colors represent different metabolic pathways. A complete list of the annotations is provided in Table S6.

**Fig 4:**
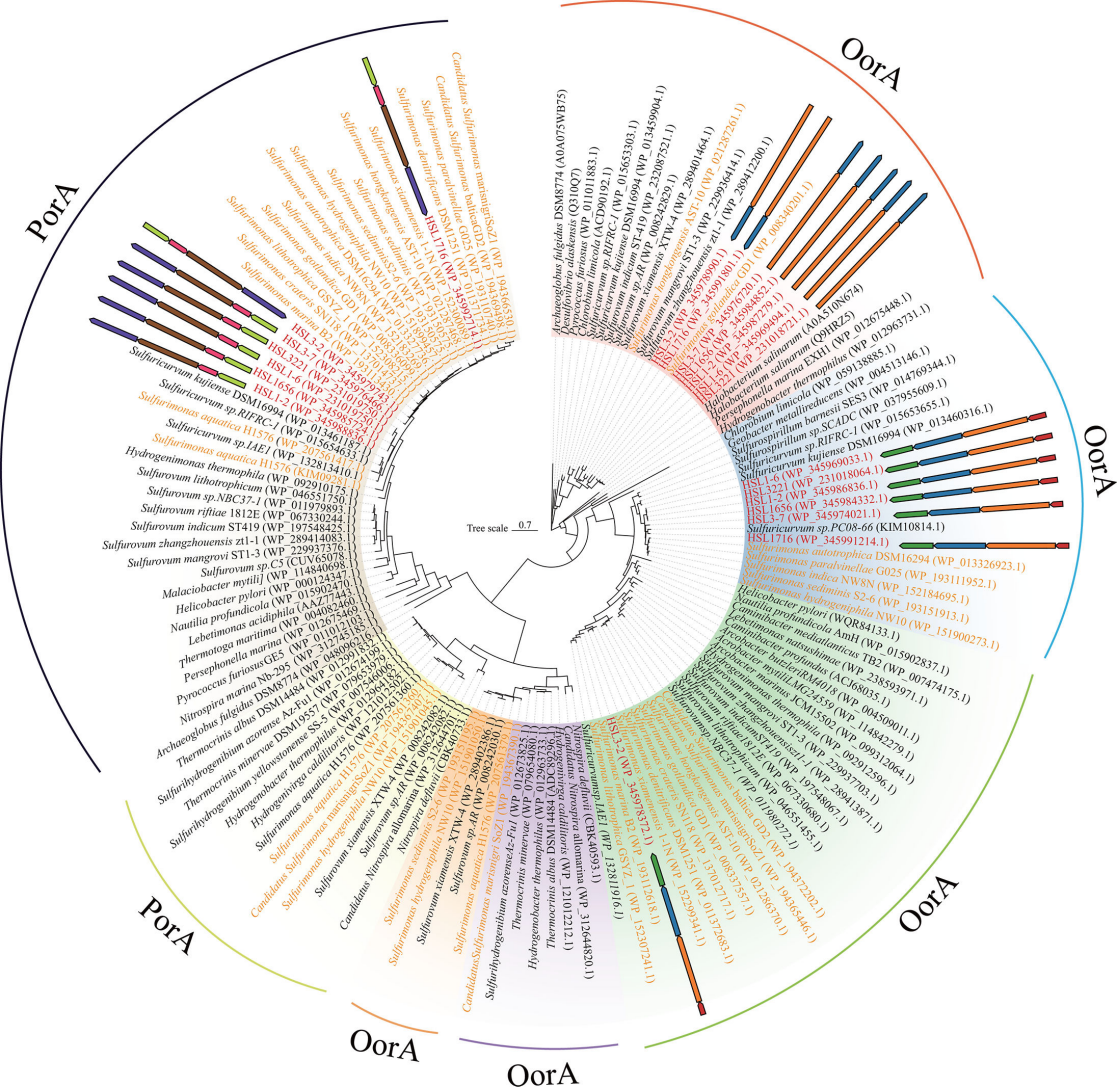
Maximum-likelihood tree based on the alpha subunits of 2-oxoglutarate:ferredoxin oxidoreductase (OorA) and pyruvate:ferredoxin oxidoreductase (PorA) involved in the rTCA cycle of seven strains. The text highlighted in red represents our strains, and orange represents the genus *Sulfurimonas*. Bootstrap values shown on each node are based on a total of 1,000 bootstrap replicates. Branch node values below 50% are not shown. Bar, 0.7 substitutions per position.

All seven isolated strains relied on chemoautotrophic metabolisms, acting as primary producers and thus providing resources for the growth of other microbes living in mangrove sediments. Specifically, the seven strains contained all genes for the reverse tricarboxylic acid (rTCA) cycle, including the key genes encoding ATP-dependent citrate lyase (Acl), pyruvate:ferredoxin oxidoreductase (Por), and 2-oxoglutarate:ferredoxin oxidoreductase (Oor) (Fig. 3; Table S6). As Por and Oor are highly sensitive to O_2_, the rTCA cycle is mainly found in diverse lineages of microaerobes and anaerobes such as *Pseudomonadota*, *Campylobacterota*, and *Aquificales* (24). In the Aquificales member *Hydrogenobacter thermophilus*, ﬁve-subunit O_2_-tolerant forms of Oor and Por mainly support aerobic growth (up to 40%), whereas O_2_-sensitive two-subunit forms are used under anaerobic conditions (25). All seven isolated strains harbored two- and four-subunit versions of Oor and four subunits of Por, while in *Sulfurimonas*, almost all strains contained only a four-subunit version of Oor and Por (Table S6). The phylogenetic analysis indicated that the two-subunit Oor of the seven strains clustered with those of thermophilic bacteria and archaea such as *Pyrococcus furiosus* and *‌Archaeoglobus fulgidus* as well as green sulfur bacteria (Fig. 4), pointing to a possible origin through horizontal gene transfer (26). Notably, these Oors of seven strains with two subunits were clustered with those from *H. thermophilus*, indicating that they could play a similar function under anaerobic conditions. Furthermore, the four-subunit Oors of seven strains were distinct from the Oor from *H. thermophilus* and clustered together with diverse microaerobic or anaerobic taxa of *Campylobacteria* (Fig. 4). Strains in *Thiomicrolovo* and *Sulfonitrofixus*, as well as *H. sulfurireducens* HSL1716^T^ with four subunits of Oor, were clustered with that of the genus *Sulfuricurvum* and vent *Sulfurimonas* strains. *H. mobilis* HSL3-2^T^ was clustered with non-vent *Sulfurimonas* strains and other members of *Campylobacteria* (Fig. 4). These results indicated that this Oor with four subunits was conserved in *Campylobacteria* and had a differential evolution in accordance with the distinct habitats. Furthermore, the Pors with four subunits of seven strains were phylogenetically far from the Por from *H. thermophilus* and clustered with functionally characterized four-subunit Pors of thermophiles *P. furiosus*, *A. fulgidus*, *Thermotoga maritima*, and nitrite-oxidizing bacterium *Nitrospira marina* (Fig. 4). A previous study has indicated that the putative four-subunit Por in *N. marina* exhibited the highest abundance under low O_2_ conditions (27), and thus the four-subunit Por in seven strains may also play a role under O_2_-limiting conditions.

#### Sulfur metabolism

**Fig 5:**
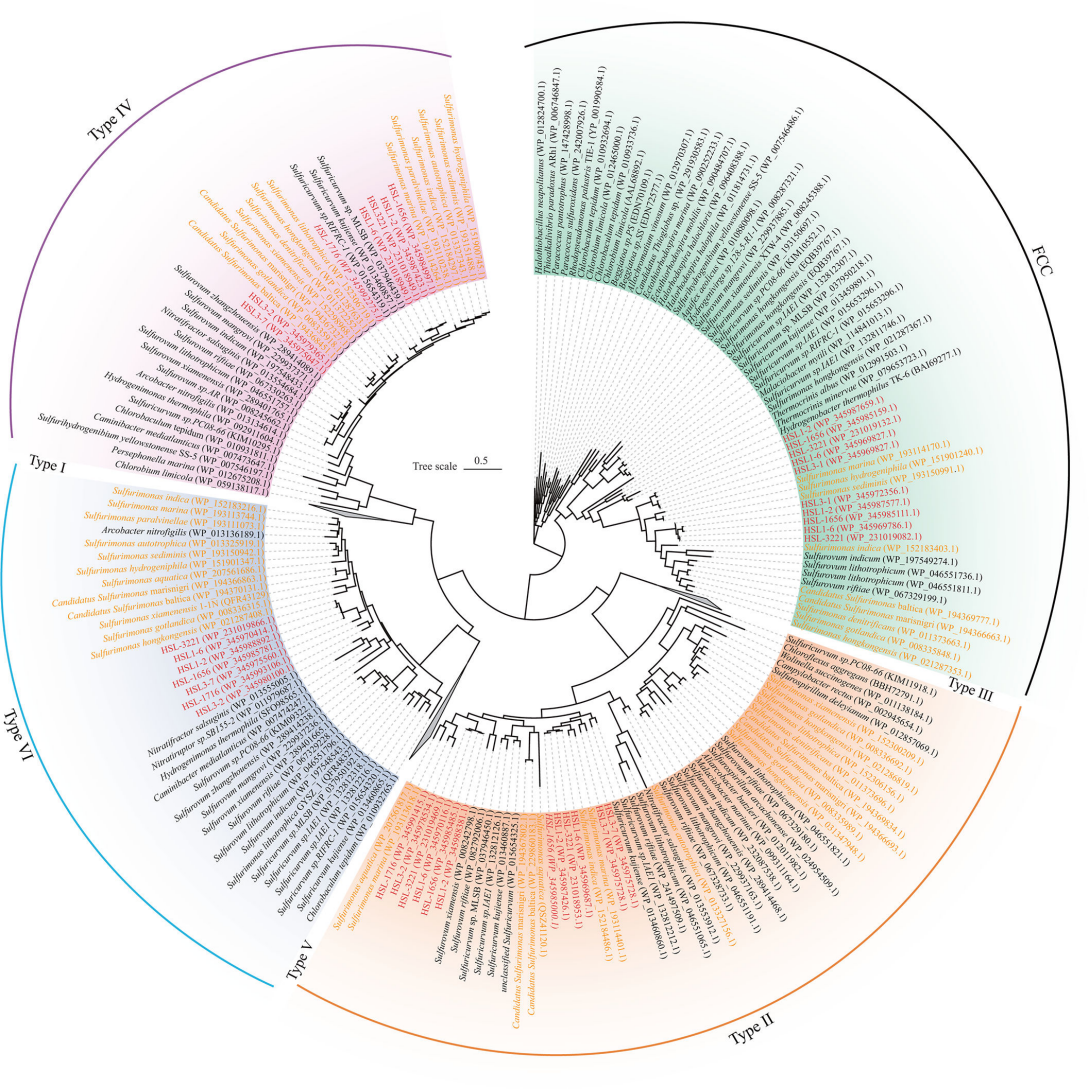
Maximum-likelihood tree of the beta subunit of flavocytochrome c sulfide dehydrogenase (Fcc) and sulfide:quinone oxidoreductase involved in sulfide oxidation of seven strains. The text highlighted in red represents our strains, and orange represents the genus *Sulfurimonas*. Bootstrap values shown on each node are based on a total of 1,000 bootstrap replicates. Branch node values below 50% are not shown. Bar, 0.5 substitutions per position.

Sulfur metabolism is a hallmark of the functional roles of *Campylobacteria* (28). For thiosulfate oxidation, all four *Thiomicrolovo* strains and *H. mobilis* HSL3-2^T^ possessed two separate sox clusters, i.e., *soxABXY*_*1*_*Z*_*1*_ and *soxCDY*_*2*_*Z*_*2*_. *S. jiaomeiensis* HSL3-7^T^ and *H. sulfurireducens* HSL-1716^T^ possessed only *soxABXY*_*1*_*Z*_*1*_ or *soxCDY*_*2*_*Z*_*2*_, respectively (Fig. 3). While in *Sulfurimonas*, only *Sulfurimonas xiamenensis* 1-1N^T^ and *Sulfurimonas lithotrophica* GYSZ_1^T^ possessed the *soxCDY*_*2*_*Z*_*2*_ cluster, no pure cultures of *Sulfurimonas* contained only the *soxABXY*_*1*_*Z*_*1*_ cluster (Fig. 3). The absence of *soxCD* indicates an incomplete thiosulfate oxidation, resulting in the generation of intermediate sulfur product, as confirmed in other members from green sulfur bacteria (phylum *Chlorobi*) and purple sulfur bacteria (class *Gammaproteobacteria*) (29). A previous study has shown that *Sulfurimonas* strains possessing only the *soxCDY*_*2*_*Z*_*2*_ cluster are incapable of utilizing thiosulfate but exhibit the ability of biological S^0^ oxidation (30), implying that strain HSL-1716^T^ might perform a similar metabolism. For sulfide oxidation, all seven strains contained flavocytochrome c sulfide dehydrogenase (Fcc) and sulfide:quinone oxidoreductase (Sqr) with variation in numbers (Table S6). Four *Thiomicrolovo* strains contained two copies of Fcc, and other strains in *Sulfonitrofixus* and *Hydrocurvibacter* lacked Fcc, which was different from *Sulfurimonas* spp. that had only one copy (Table S6). Phylogenetic analysis showed that Fcc with two copies grouped into distinct branches and further clustered together with those of *Sulfurimonas*, *Sulfurovum*, Aquificales, and thermophilic archaea (Fig. 5). Furthermore, seven strains contained diverse types of Sqr, including types II, IV, and VI. Both *Hydrocurvibacter* strains contained one copy of type II Sqr, while all the other strains in *Thiomicrolovo* and *Sulfonitrofixus* contained two copies (Table S6). Phylogenetic analysis indicated that type II Sqr of seven strains grouped into different clusters with diverse *Campylobacteria* members (Fig. 5). Moreover, all strains contained one copy of type IV and VI Sqrs, which was also conserved in *Sulfurimonas* (Fig. 3), likely to cope with diﬀerent sulﬁde concentrations under distinct environmental conditions (26). Phylogenetic analysis further showed that type IV Sqr of seven strains was clustered with the members from green sulfur bacteria and Aquificales, and type VI Sqr of seven strains grouped together with diverse *Campylobacteria* (Fig. 5). Additionally, all seven strains contained polysulfide reductase (Psr) and thiosulfate reductase (Phs), which could be involved in sulfur reduction or disproportionation (31). Moreover, although the ability of *S. jiaomeiensis* HSL3-7^T^ to perform sulfate reduction has been confirmed in this study, none of the known genes involved in dissimilatory sulfate reduction were found (Table S6), implying the existence of an unrevealed sulfate reduction pathway. Further studies based on transcriptomics and proteomics will be needed to elucidate this metabolism.

#### Hydrogen oxidation

**Fig 6:**
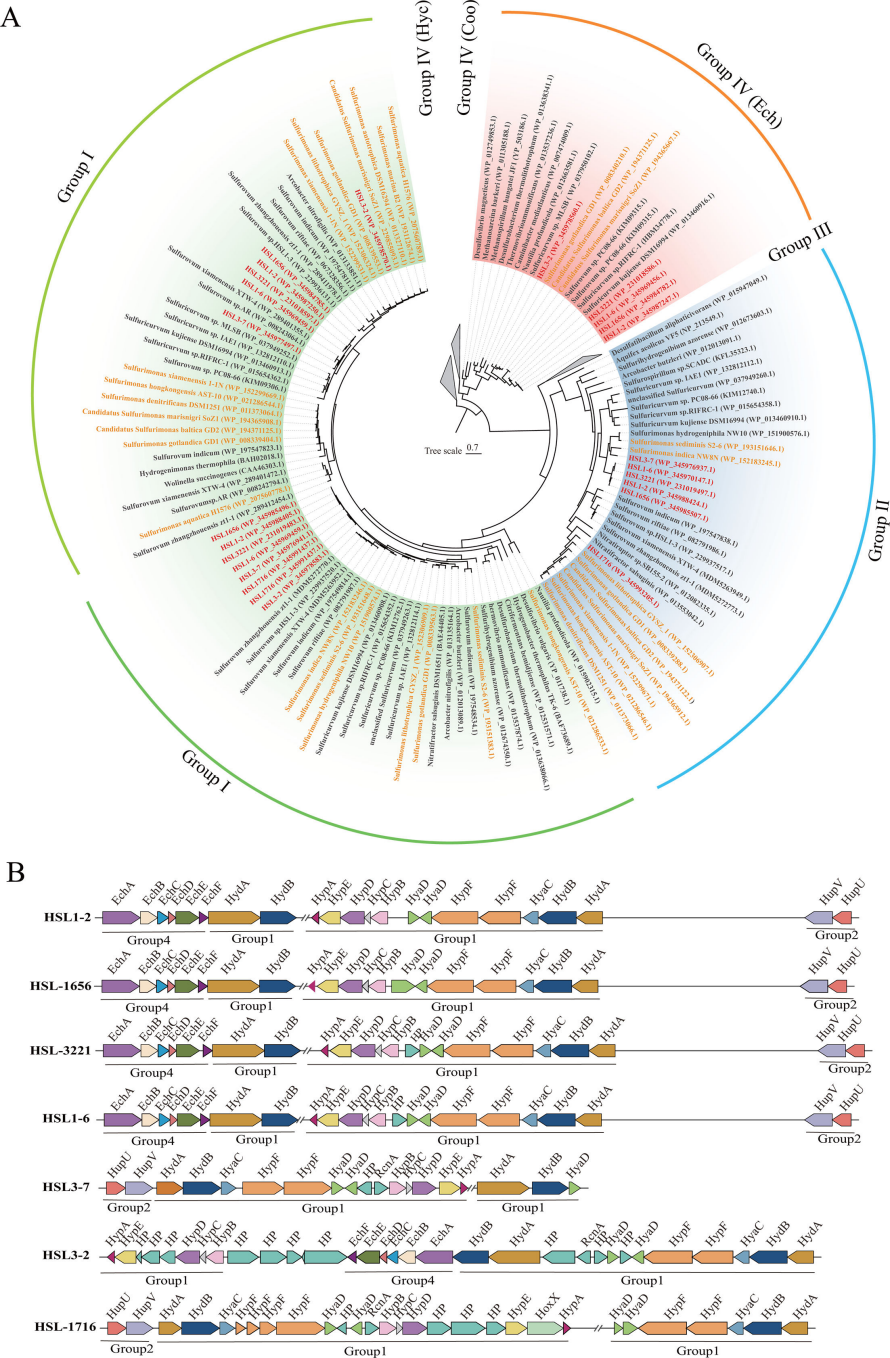
Maximum-likelihood tree based on large subunit sequences of hydrogenase from seven strains (**A**) and the schematic diagram of the hydrogenase gene cluster (**B**)**.** The text highlighted in red represents our strains, and orange represents the genus *Sulfurimonas*. Bootstrap values shown on each node are based on a total of 1,000 bootstrap replicates. Branch node values below 50% are not shown. Bar, 0.7 substitutions per position.

The oxidation of H_2_ can also be exploited as a possible energy source by seven strains since they all encoded a group I [NiFe]-hydrogenase (Fig. 3), which was consistent with the phenotypic data. The energy is gained by the proton motive force generated across the periplasmic membrane from the oxidation of H_2_ to protons and electrons (32). All seven strains had two copies of group I hydrogenases, which may reflect the ability to function at different hydrogen concentrations, as previously reported in *Sulfurimonas* and other *Campylobacteria* (33). Phylogenetic analysis showed that group I hydrogenases of seven strains grouped into different clusters with diverse *Campylobacteria* members (Fig. 6A). Except for *H. mobilis* HSL3-2^T^, the other six strains harbored group II hydrogenases, possibly involved in hydrogen sensing or energy conversion at low hydrogen concentrations (32). Phylogenetic analysis showed that four strains in *Thiomicrolovo* clustered into a single branch; *S. jiaomeiensis* HSL3-7^T^ clustered with the vent strains of *Sulfurimonas*, whereas *H. sulfurireducens* HSL-1716^T^ clustered with the non-vent *Sulfurimonas* strains (Fig. 6A). Except for *S. jiaomeiensis* HSL3-7^T^ and *H. sulfurireducens* HSL-1716^T^, the other five strains harbored a group IV hydrogenase, Ech, an energy-converting hydrogenase typically present in pelagic *Sulfurimonas* spp. like *Sulfurimonas gotlandica* GD1^T^, *Sulfurimonas marisnigri* SoZ1^T^, and *Sulfurimonas baltica* GD2^T^ (Fig. 3). Phylogenetic analysis indicated that Ech of the five strains was clustered with thermophilic archaea and Aquiﬁcales (Fig. 6A), implying the occurrence of horizontal gene acquisitions (26). The Ech hydrogenase has been well characterized in the archaeon *Methanosarcina barkeri*, where it catalyzes hydrogen formation through reduced ferredoxin (34). Likewise, these strains identified here might have the potential to internally recycle this hydrogen as an energy source, as previously described in nitrogen-fixing microbes from deep-sea cold seeps (35). Moreover, the gene cluster organization of hydrogenases in seven strains demonstrated that *Thiomicrolovo* strains had the same arrangement of groups I, II, and IV, whereas *Sulfonitrofixus* and *Hydrocurvibacter* had a different arrangement (Fig. 6B). Although we did not have a direct quantification of the H_2_ level within mangrove sediments, H_2_ has been reported as one of the common electron donors and thus may represent an important energy source for chemoautotrophs, which is produced from the fermentation of macromolecules in deeper sediments (36).

#### Nitrogen metabolism

All seven strains possessed the complete nitrogen fixation pathway catalyzed by the nitrogenase encoded by nifHDK, whereas in *Sulfurimonas*, only *S. marisnigri* SoZ1^T^, *S. lithotrophica* GYSZ_1^T^, *S. baltica* GD2^T^, and *S. marina* B2^T^ possessed this pathway (Fig. 3). Mangrove sediments are widely recognized as nitrogen-limited ecosystems due to tidal fluctuation and high denitrification rates, and nitrogen-ﬁxing bacteria are considered to play a key role in nitrogen amendment (37, 38). Thus, the strains isolated in this study containing nitrogenase genes are of competitive advantage in mangrove sediments. Interestingly, all seven strains contained the incomplete denitrification pathway or even lacked this pathway (Fig. 3). *T. zhangzhouensis* HSL1-2^T^, *T. subterrani* HSL-1656^T^, and *H. sulfurireducens* HSL-1716^T^ contained only the nitrate reductase encoded by NapABDGHL and nitric oxide reductase encoded by NorBC, whereas the nitrite reductase encoded by NirKS and nitric oxide reductase encoded by NosZ were absent. *T. sulfuroxydans* HSL-3221^T^, *T. immobilis* HSL1-6^T^, *S. jiaomeiensis* HSL3-7^T^, and *H. mobilis* HSL3-2^T^ totally lacked this pathway. These genome analysis results were consistent with the phenotypic data. However, in *Sulfurimonas*, the complete denitrification pathway was quite common (Fig. 3). Sulfur oxidation coupled with nitrate reduction is considered the primary process carried out by members of *Sulfurimonas* in coastal marine sediments and the Baltic Sea redoxcline (39), whereas this process does not occur in most of the new isolates, implying that these isolated strains in mangrove sediments likely either cooperate with other denitrifying microbes or use other electron acceptors.

#### Oxygen respiration

Since the upper layers of the mangrove sediments studied were not strictly anoxic, an oxidative respiratory chain associated with terminal oxidases for different affinities to oxygen was identified in these isolated strains. Specifically, *T. subterrani* HSL1656^T^, *T. sulfuroxydans* HSL-3221^T^, *T. immobilis* HSL1-6^T^, and *H. sulfurireducens* HSL1716^T^ contained diverse respiratory oxygen reductases encoding the *aa*_*3*_- and *cbb*_*3*_-type cytochrome c oxidases (*coxABCD* and *ccoNOQP*, respectively) and cytochrome bd ubiquinol oxidase (cydAB) (Fig. 3). *T. zhangzhouensis* HSL1-2^T^ and *S. jiaomeiensis* HSL3-7^T^ contained genes ccoNOQP and *cydAB*, while *H. mobilis* HSL3-2^T^ harbored only *ccoNOQP*. In contrast, all *Sulfurimonas* strains encoded the *ccoNOQP*; no pure culture contained the complete cluster of *coxABCD*, and only *Sulfurimonas hydrogeniphila* NW10^T^, *Sulfurimonas sediminis* S2-6^T^, *S. xiamenensis* 1-1N^T^, and *S. marina* B2^T^ harbored *cydAB* (Table S6). It is known that the *cbb*_*3*_-type and bd oxidases are high-afﬁnity terminal oxygen reductases capable of functioning under microoxic to anoxic conditions, whereas the low-oxygen afﬁnity *aa*_*3*_-type oxidase is induced under oxic conditions (40). Thus, the redundant terminal oxidases in these isolated strains might reflect their respiratory flexibility in the fluctuating environments of mangrove sediments. In fact, growth experiments showed that the cultured strains, especially *T. zhangzhouensis* HSL1-2^T^ and *T. subterrani* HSL-1656^T^, could grow at the O_2_ concentrations up to 20% (Table 1). Similar observations have been found in some vent isolates, such as *Sulfurimonas autotrophica*, which may be attributed to the antioxidation systems within them (24, 41).

### Metatranscriptomic analysis unveils *in situ* metabolic activities of new isolates

**Fig 7:**
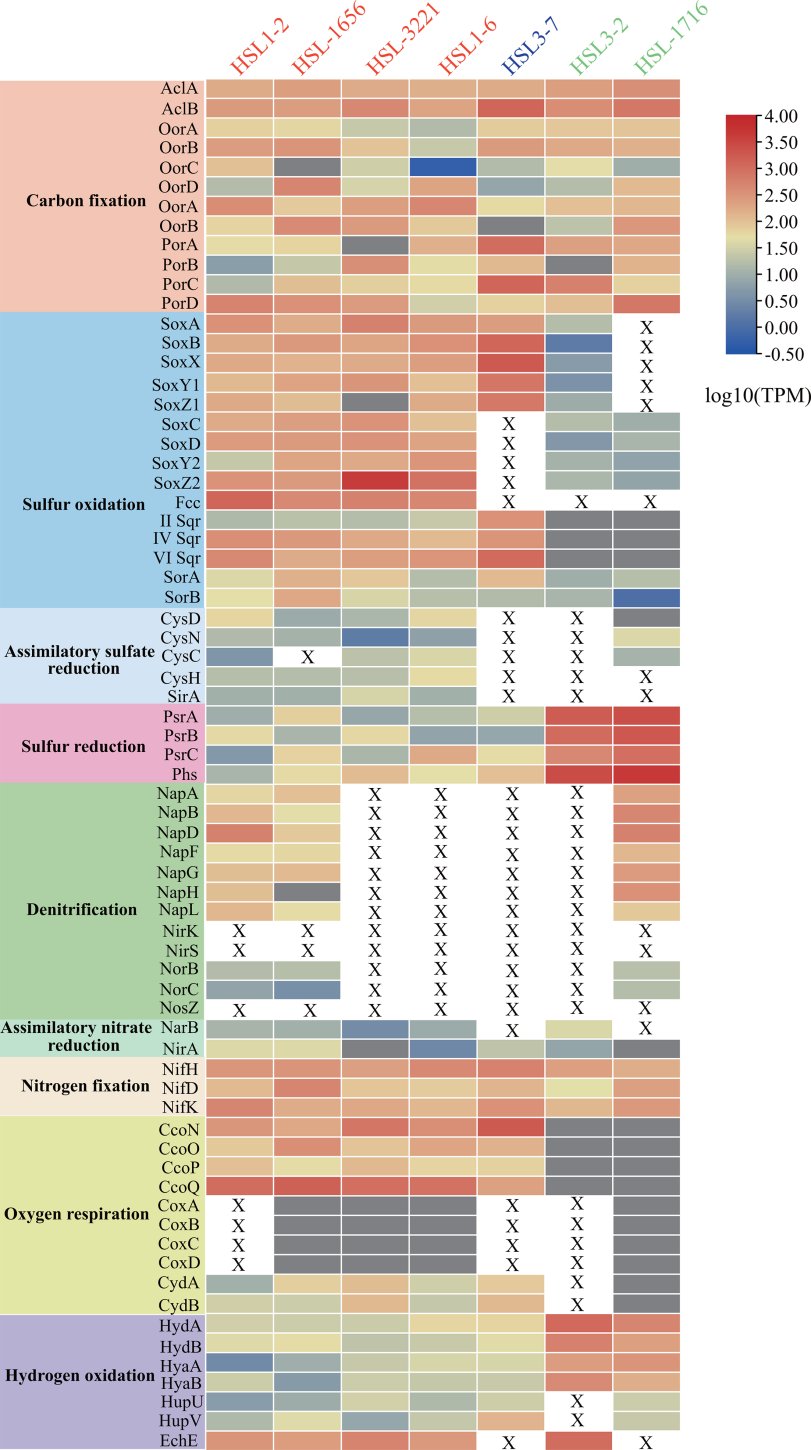
Heatmaps of the gene expression associated with energy metabolism in seven strains within mangrove sediments. The expression of each gene in each strain is the sum of the data from all sediment layers, and transition from blue to red represents increased expression. Transcripts per million (TPM) is presented in log10 scale, and the detailed TPM values at each layer are listed in Table S7.

In order to understand the *in situ* metabolizing activities of the seven new isolates of *Campylobacteria*, metatranscriptomic analysis of 10 mangrove sediment samples of 0–20 cm layers was conducted. The results showed that the key genes of the rTCA cycle encoding Acl, Oor, and Por with two or four subunits were actively expressed (Fig. 7), indicating that the rTCA cycle was the main pathway for autotrophic growth. For *Thiomicrolovo* strains, transcripts of *soxABXY*_*1*_*Z*_*1*_ and *soxCDY*_*2*_*Z*_*2*_ were expressed with transcripts per million (TPM) values of >2,000, mainly occurring at 10–20 cm sediment layers (Table S7). The expression of *soxCD* indicates a complete oxidation of one molecule of thiosulfate into two molecules of sulfate (29), suggesting that they have a contribution to the production of sulfate *in situ*. For the genus *Sulfonitrofixus*, *soxABXY*_*1*_*Z*_*1*_ was actively expressed with TPM values of >1,000 at 8–20 cm layers (Table S7), indicating that it could partly oxidize S_2_O_3_^2−^ with element sulfur accumulation *in situ*. Our results highlight the potential ecological role of extracellular S^0^ produced by isolated strains in mangrove sediments. For *Hydrocurvibacter*, the transcripts of *soxABXY*_*1*_*Z*_*1*_ and *soxCDY*_*2*_*Z*_*2*_ in strain HSL3-2^T^ and *soxCDY*_*2*_*Z*_*2*_ in strain HSL-1716^T^ were not expressed or expressed at very low levels (<10 TPM, Fig. 7), indicating that these strains may gain energy by other metabolic pathways. For sulfide oxidation, the transcripts of Fcc and type IV and VI Sqr in *Thiomicrolovo* were expressed at varying degrees with values of 22.98–478.82 TPM, suggesting that these strains could oxidize sulfide by both pathways (Fcc and Sqr) *in situ*. Thus, we propose that in the oxic and high-sulfide niches of mangrove sediments, sulfide oxidation may be promoted by the Sqr enzyme; in the low-oxygen and low-sulfide areas, Fcc might be used to perform the sulfur oxidation, as described previously in shallow-water gas vent and cold seeps (42, 43). Furthermore, *S. jiaomeiensis* HSL3-7^T^ exhibited differential expression levels of type II, IV, and VI Sqrs, and these transcripts were not expressed in *Hydrocurvibacter* (Fig. 7), indicating that sulfide oxidation may be more important for *Sulfonitrofixus*. The expression of hydrogen oxidization-associated gene *hydB* in *Thiomicrolovo* and *Sulfonitrofixus* was insignificant (1.05–29.41 TPM) compared with their sulfur oxidation genes, suggesting that reduced sulfur compounds are important energy sources for both genera. While in *Hydrocurvibacter*, *hydB* was actively expressed with 46.03–327.07 TPM, suggesting that hydrogen oxidation may be an important energy source for them. Moreover, group II hydrogenases were transcribed only at lower levels (1.22–48.43 TPM) in three genera, while group IV Ech hydrogenase was actively expressed at different levels in five strains (45.40–428.08 TPM, Fig. 7). These results indicate that these strains with Ech could catalyze hydrogen formation, thus playing a role in the nutrient cycling in mangrove sediments. Previous studies have shown that hydrogen is also an alternative energy source for Gammaproteobacteria in marine surface sediments (44). In fact, *Sulfurimonas denitrificans* grows more efficiently with hydrogen than with thiosulfate, when the electron acceptor of nitrate is limiting (45). Thus, hydrogen oxidation could be a hitherto overlooked energy source for microbes in mangrove sediments.

In addition to carbon, sulfur, and hydrogen metabolism, the transcripts of NifHDF, encoding nitrogenase, were expressed in *T. sulfuroxydans* HSL-3221^T^, *T. immobilis* HSL1-6^T^, *S. jiaomeiensis* HSL3-7^T^, *H. mobilis* HSL3-2^T^, and *H. sulfurireducens* HSL-1716^T^ (up to 314.41 TPM, Fig. 7), illustrating that these strains could actively fix nitrogen to synthesize organic nitrogen for growth. These findings highlight the important ecological role of these novel *Campylobacteria* spp. in supplying nitrogen for the microbiome community in the nitrogen-limited mangrove sediments. Furthermore, the expressions of NapABDGHL in *T. zhangzhouensis* HSL1-2^T^, *T. subterrani* HSL-1656^T^, and *H. sulfurireducens* HSL-1716^T^ (up to 267.54 TPM) were detected, and the gene encoding NorBC in the three strains was not expressed or expressed at lower levels (1.02–9.79 TPM) (Table S7), suggesting that the product of denitrification was nitrite, not nitrogen, which is consistent with the previous study in other mangrove sediments (46). The periplasmic nitrate reductase (Nap) has a high affinity for nitrate and may be advantageous in a nitrate-limited environment, like Olkiluoto groundwater, Finland (47). Similarly, the nitrate concentration in mangrove sediments was generally low (37), and thus the isolated strains encoding and expressing Nap may represent an adaptation to low nitrate environments. For oxygen respiration, the transcripts for CcoNOQP were found in *Thiomicrolovo* (11.54–931.26 TPM); CydAB was expressed at a lower level (1.64–63.80 TPM); and CoxABCD was not expressed at all. For *Sulfonitrofixus*, CcoNOQP was actively expressed (up to 1046.44 TPM), and CydAB was expressed at a much lower level (3.74–40.42 TPM, Table S7). The relatively high expression of the CcoNOQP genes is consistent with oxygen reduction in the suboxic mangrove sediments (48), suggesting that the *cbb*_*3*_-type terminal oxidase might enable these strains to continue aerobic respiration at low O_2_ concentrations. Additionally, these enzymes possibly play a role in oxygen scavenging to prevent poisoning (49). However, CcoNOQP from *H. mobilis* HSL3-2^T^, along with CcoNOQP, CoxABCD, and CydAB from *H. sulfurireducens* HSL-1716^T^, was not expressed (Fig. 7). In contrast, both *Hydrocurvibacter* strains expressed the genes encoding Psr and Phs in the deeper layers except the surface (up to 1762.09 TPM), whereas these genes were expressed at very low or undetectable levels in *Thiomicrolovo* and *Sulfonitrofixus* (Table S7), suggesting that *Hydrocurvibacter* strains may gain energy from sulfur reduction or disproportionation *in situ*. These results indicate that the members of three potentially new genera from *Campylobacteria* could utilize diverse chemical energy sources for growth in mangrove sediments, adapting to the habitat via special metabolic mechanisms.

### The relative abundance of novel isolates in mangrove sediments and the wide distribution in the global oceans

**Fig 8:**
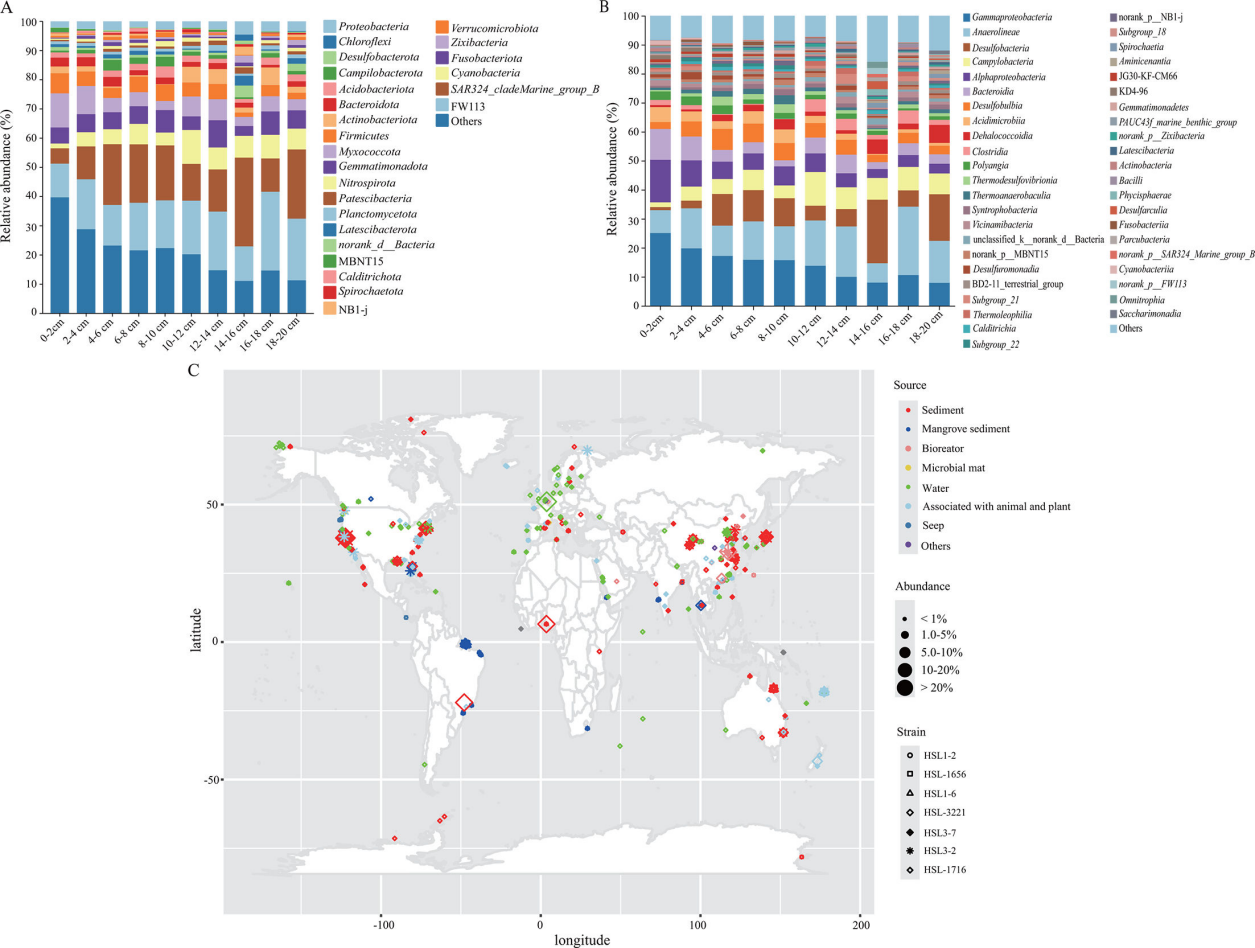
Taxonomic distribution based on 16S rRNA gene sequence and global distribution pattern of all seven strains**.** (**A**) The microbial compositions among the 10 mangrove sediments of 0–20 cm layers at the phylum level. (**B**) The microbial compositions among the 10 mangrove sediments at the class level. For each plot, all taxa present in a relative abundance of ≥1.0% in at least one of the samples are shown. (**C**) Global distribution of all seven strains in this study. Bacteria of three new genera are widely distributed in various environments. Their metadata were all retrieved from environmental samples.

The compositions of the microbial communities within 0–20 cm mangrove sediments of Jiulong River estuary in Fujian Province, China, are investigated. At the phylum level, the four most dominant community members were Proteobacteria (13.5%–39.8%), Chloroflexi (11.6%–26.9%), Desulfobacterota (5.2%–30.4%), and Campylobacterota (1.6%–11.6%) (Fig. 8A). Similarly, at the class level, the four most dominant taxa were Gammaproteobacteria (7.9%–25.1%), Anaerolineae (6.6%–23.6%), Desulfobacteria (1.1%–21.9%), and *Campylobacteria* (1.6%–11.6%) (Fig. 8B). These results indicate that *Campylobacteria* spp. are one of the key communities in these mangrove sediments, implying their important roles *in situ*, similar to other chemosynthesis-based habitats (7). A total of 20 ASVs (97% similarity threshold with isolated strains) were retrieved in all samples, with ASV3 and ASV19 being predominant (the relative abundances >1%) (Table S8 and S9). Specifically, *T. zhangzhouensis* HSL1-2^T^ and *T. subterrani* HSL-1656^T^ exhibited the highest sequence identity of 99.01% with ASV4608, accounting for less than 0.01% read abundance at 18–20 cm sediment layers (Table S9). They also shared a sequence similarity of 98.02% with ASV19, which was one of the most predominant members with an abundance of 0.11%–1.03% at 0–20 cm layers. *T. sulfuroxydans* HSL-3221^T^ and *T. immobilis* HSL1-6^T^ demonstrated the highest sequence identity of 99.01% with ASV5457, accounting for 0.05% relative abundance at 10–12 cm layers. Both strains also showed the sequence similarities of 98.52% and 97.03% with ASV19 and ASV3, respectively (Table S8). ASV3 was another predominant member in mangrove sediments, with values of 1.01%–2.46% at 4–10, 14–16, and 18–20 cm layers (Table S9). Furthermore, *S. jiaomeiensis* HSL3-7^T^ exhibited the highest sequence identities of 99.99%, 99.26%, and 99.01% with ASV23, ASV4042, and ASV3, respectively, and it also had 97.56% sequence similarity with ASV19. The relative abundance of ASV23 was 0.06%–0.45% at the sediment layers of 6–20 cm, and the abundance of ASV4042 was 0.08%–0.12% at 2–4, 4–6, 6–8 and 18–20 cm layers. In contrast, *H. mobilis* HSL3-2^T^ and *H. sulfurireducens* HSL-1716^T^ shared the highest sequence identities with ASV4663, which was merely 96.29% (Table S8). Thus, these results indicated that all four *Thiomicrolovo* strains and *S. jiaomeiensis* HSL3-7^T^ represented the most predominant members in *in situ* mangrove sediments, corresponding to the predominant members represented by ASV3 and ASV19.

Furthermore, we analyzed the global distribution pattern of seven isolated strains by submitting the 16S rRNA gene sequences on the integrated microbial next-generation sequencing (IMNGS) platform (50). The result indicated that they were widely distributed in various ecosystems, including seawater columns (0.01%–1.88%), marine sediments (0.01%–32.28%), mangrove sediments (0.01%–2.66%), deep-sea hydrothermal vents (0.00%–0.36%), microbial mats (0.01%–0.09%), cold seeps (0.01%–0.34%), seagrass (0.01%–0.06%), bioreactors (0.00%–84.14%), and animal intestines (0.01%–0.28%) (Fig. 8C; Table S10). These habitats are always hypoxic and anoxic environments due to the intense microbial activities consuming oxygen and the enclosed nature of the environments (51). In general, the seven strains exhibited relatively high abundances in the habitats including bioreactors, marine sediments, and mangrove sediments, with the abundances of 1.02%–84.14%, 1.02%–32.28%, and 1.07%–2.66%, respectively, indicating that they may play more important roles in these ecological systems. Among the seven isolates, *H. sulfurireducens* HSL1716^T^ was the most widely distributed and found in a variety of different habitats, and only *T. zhangzhouensis* HSL1-2^T^ and *H. sulfurireducens* HSL1716^T^ were detected at deep-sea hydrothermal vents (Table S10). The wide distribution of the seven new strains in various ecosystems demonstrates their adaptability to different habitats and highlights their important ecological roles.

### Conclusions

In this study, we reported the isolation of seven pure cultures from the class *Campylobacteria* and systematically analyzed the characterization of their genomes and *in situ* metabolic activity in mangrove sediments. These isolates represented seven potentially new species belonging to three new genera, which greatly expands our understanding of the species diversity within the class *Campylobacteria*. Diverse metabolic traits were revealed in these novel isolates, such as various sulfur oxidation pathways, hydrogen oxidation, nitrogen fixation, sulfate reduction, and sulfur disproportionation. They also demonstrated the ability to adapt to low O_2_ conditions, such as the more efficient Oor complex for CO_2_ fixation and diverse terminal oxidases, including Cco, Cox, and Cyd for different affinities to oxygen. Metatranscriptomic analysis further confirmed their *in situ* activity and different adaptation mechanisms in mangrove sediments. Combined with their wide distributions in diverse habitats, these novel *Campylobacteria* spp. could play an important role in the biogeochemical cycling of carbon, sulfur, and nitrogen. Overall, this study promotes our understanding of the *in situ* function and ecological role of *Campylobacteria*, especially in previously overlooked mangrove sediments and other carbon-rich sediment ecosystems. Further physiological experiments involving transcriptome analysis and gene knockout techniques are necessary to elucidate the metabolism and adaptation mechanisms.

### Proposal of type names from isolated strains

#### Description of *Thiomicrolovo* gen. nov.

##### *Thiomicrolovo* (Thi·o·mi·cro·lo·vo. L. n. *sulfurium*, sulfur; Gr. adj. *mikros*, small; Gr. n. *lobos*, lobe; L. n. *ovum*, egg; N.L. n. *Thiomicrolovo*, sulfur-related small lobe-shaped or oval microorganism)

Cells are gram negative, non-motile, coccoid to oval shaped; mesophilic and aerobic; and require sea salts for growth. Growth occurs chemolithoautotrophically with reduced sulfur compounds such as thiosulfate, sulfite, sulfide, and elemental sulfur as electron donors, using CO_2_ as a carbon source. Atmospheric nitrogen could be used as the nitrogen source. The major fatty acids consist of summed features 8 and 3, C_16:0_, and C_14:0_. Members have a genomic size of around 2.51–2.56 Mb and a genomic GC content of 57.2–57.5 mol%. 16S rRNA sequencing and genome analysis revealed that the genus falls within the *Campylobacteria*.

### Description of *Thiomicrolovo zhangzhouensis* sp. nov.

#### *Thiomicrolovo zhangzhouensis* (zhang·en’sis. N.L. *fem*; adj. *zhangzhouensis*, of or pertaining to Zhangzhou, a city in Fujian, China, where the strain was isolated)

Cells are gram negative, non-motile, coccoid to oval shaped. Growth occurs at 15°C–45°C (optimum 37°C), pH 5.5–8.0 (optimum pH 7.0), and 1.0%–6.0% (wt/vol) NaCl (optimum 3.0%). Obligate chemolithoautotrophic growth occurs with thiosulfate, S^0^, and sulfide as electron donors and oxygen or nitrate as an electron acceptor. Ammonium, nitrate, and atmospheric nitrogen are utilized as nitrogen sources. Organic substrates are not utilized as carbon sources and energy sources. The major cellular fatty acids are C_16:1_ *ω*7*c*, C_18:1_ *ω*7*c*, C_16:0_, and C_14:0_.

The type strain HSL1-2^T^ (=MCCC 1A19178^T^ = JCM 36295^T^) was isolated from mangrove sediments of Jiulong River tributaries in Zhangzhou, Fujian Province, China. The DNA GC content of the type strain is 57.2 mol%.

### Description of *Thiomicrolovo subterrani* sp. nov.

*Thiomicrolovo subterrani* (suhb·tuh·reyn·ahy. Genitive noun subterrani meaning “of the subterrane,” referring to the ecosystem from which the strain was isolated)

Cells are gram negative, non-motile, coccoid to oval shaped. Growth occurs at 10°C–40°C (optimum 32°C), pH 5.0–9.0 (optimum pH 6.5), and 2.0%–4.0% (wt/vol) NaCl (optimum 2.5%). Obligate chemolithoautotrophic growth occurs with thiosulfate, sulfite, S^0^, and sulfide as electron donors and oxygen or nitrate as an electron acceptor. Ammonium, nitrate, and atmospheric nitrogen are utilized as nitrogen sources. Organic substrates are not utilized as carbon sources and energy sources. Predominant fatty acids are C_16:1_ *ω*7*c*, C_18:1_ *ω*7*c*, C_16:0_, and C_14:0_.

The type strain HSL-1656^T^ (=MCCC 1A17955^T^ = KCTC 25167^T^) was isolated from mangrove sediments of Jiulong River tributaries in Zhangzhou, Fujian Province, China. The DNA GC content of the type strain is 57.3 mol%.

### Description of *Thiomicrolovo sulfuroxydans* sp. nov.

*Thiomicrolovo sulfuroxydans* (sul·fur·oxy.dans. L. n. *sulfur*, sulfur; N.L. part. adj. *oxydans*, oxidizing; N.L. part. adj. *sulfuroxydans*, sulfur-oxidizing)

Cells are gram negative, non-motile, coccoid to oval shaped. Growth occurs at 4°C–50°C (optimum 32°C), pH 4–9 (optimum pH 7.0), and 2%–5% (wt/vol) NaCl (optimum 3%). It is strictly aerobic. Obligate chemolithoautotrophic growth occurs with thiosulfate and sulfide as electron donors and oxygen as an electron acceptor. Ammonium and nitrate are utilized as nitrogen sources. Organic substrates are not utilized as carbon sources and energy sources. Predominant fatty acids are C_16:1_ *ω*7*c*, C_18:1_ *ω*7*c*, C_16:0_, and C_14:0_.

The type strain HSL-3221^T^ (=MCCC 1A18427^T^ = KCTC 25243^T^) was isolated from mangrove sediments of Jiulong River tributaries in Zhangzhou, Fujian Province, China. The DNA GC content of the type strain is 57.3 mol%.

### Description of *Thiomicrolovo immobilis* sp. nov.

*Thiomicrolovo immobilis* (im·mo’bi·lis. L. fem. adj. *immobilis*, immobile, immovable, pertaining to a feature of the type strain)

Cells are gram negative, non-motile, coccoid to oval shaped. Growth occurs at 4°C–45°C (optimum 35°C), pH 4.5–9.0 (optimum pH 6.0), and 1.0%–4.0% (wt/vol) NaCl (optimum 2.0%). It is strictly aerobic. Obligate chemolithoautotrophic growth occurs with thiosulfate and sulfide as electron donors and oxygen as an electron acceptor. Ammonium and nitrate are utilized as nitrogen sources. Organic substrates are not utilized as carbon sources and energy sources. Predominant fatty acids are C_16:1_ *ω*7*c*, C_18:1_ *ω*7*c*, C_16:0_, and C_14:0_.

The type strain HSL1-6^T^ (=MCCC 1A18693^T^ = KCTC 25544^T^) was isolated from mangrove sediments of Jiulong River tributaries in Zhangzhou, Fujian Province, China. The DNA GC content of the type strain is 57.5 mol%.

### Description of *Sulfonitrofixus* gen. nov.

*Sulfonitrofixus* (Sul·fo·ni·tro·fix’us. N.L. n. *sulfur*, sulfur; L. n. *nitro* (from nitrum), soda ash, saltpeter; L. n. *fixus*, fixed, fastened; N.L. masc. n. *Sulfonitrofixus*, sulfur and nitrogen fixing)

Cells are gram negative, non-motile, rod shaped; mesophilic and aerobic; and require sea salts for growth. Growth occurs chemolithoautotrophically with hydrogen, sulfide, and thiosulfate as electron donors, using CO_2_ as a carbon source. Atmospheric nitrogen could be used as the nitrogen source. The major fatty acids consist of summed features 8 and 3, C_16:0_, and C_14:0_. Member has a genomic size of around 2.87 Mb and a genomic GC content of 48.7 mol%. 16S rRNA sequencing and genome analysis revealed that the genus falls within the *Campylobacteria*.

### Description of *Sulfonitrofixus jiaomeiensis* sp. nov.

#### *Sulfonitrofixus jiaomeiensis* (jia·o·mei·en’sis. N.L. fem. adj. *jiaomeiensis*, pertaining to the Jiaomei town in Zhangzhou, from where the type strain was isolated)

Cells are gram negative, non-motile, and rod shaped. Growth occurs at 15°C–45°C (optimum 37°C), pH 4.5–8.5 (optimum pH 7.0), and 2.0%–4.0% (wt/vol) NaCl (optimum 2.5%). Obligate chemolithoautotrophic growth occurs with H_2_, thiosulfate, and sulfide as electron donors and S^0^, oxygen, or sulfate as an electron acceptor. Ammonium, nitrate, and atmospheric nitrogen are utilized as nitrogen sources. Organic substrates are not utilized as carbon sources and energy sources. Predominant fatty acids are C_16:1_ *ω*7*c*, C_18:1_ *ω*7*c*, C_16:0_, and C_14:0_.

The type strain HSL3-7^T^ (=MCCC 1A18694^T^ = KCTC 25546^T^) was isolated from mangrove sediments of Jiulong River tributaries in Zhangzhou, Fujian Province, China. The DNA GC content of the type strain is 48.7 mol%.

### Description of *Hydrocurvibacter* gen. nov.

#### *Hydrocurvibacter* (Hy·dro·cur·vi.bac’ter. N.L. neut. n. *hydrogenium*, hydrogen; L. adj. *curvus*, curved; N.L. masc. n. *bacter*, rod; N.L. masc. n. *Hydrocurvibacter*, hydrogen-utilizing curved rod)

Members are gram negative and rod shaped or slightly curved; variably motile; mesophilic and aerobic; and require sea salts for growth. Growth occurs chemolithoautotrophically with hydrogen, sulﬁde, elemental sulfur, and thiosulfate as electron donors, using CO_2_ as a carbon source. Atmospheric nitrogen could be used as the nitrogen source. The major fatty acids consist of summed features 8 and 3 and C_16:0_. Members have a genomic size of around 2.24–2.26 Mb and a genomic GC content of 40.4–41.7 mol%. 16S rRNA sequencing and genome analysis revealed that the genus falls within the *Campylobacteria*.

### Description of *Hydrocurvibacter mobilis* sp. nov.

#### *Hydrocurvibacter mobilis* (mo’bi·lis. L. masc. adj. *mobilis*, motile)

Cells are gram negative, rod shaped, and motile with a polar flagellum. Growth occurs at 10°C–45°C (optimum 37°C), pH 5.4–8.6 (optimum pH 6.1), and 1.0%–5.0% (wt/vol) NaCl (optimum 3.0%). It is strictly aerobic. Obligate chemolithoautotrophic growth occurs with H_2_, thiosulfate, S^0^, and sulfite as electron donors and S^0^ or oxygen as the electron acceptor. Ammonium, nitrate, and atmospheric nitrogen are utilized as nitrogen sources. Organic substrates are not utilized as carbon sources and energy sources. Predominant fatty acids are C_16:1_ *ω*7*c*, C_18:1_ *ω*7*c*, and C_16:0_.

The type strain HSL3-2^T^ (=MCCC 1A19147^T^ = KCTC 25545^T^) was isolated from mangrove sediments of Jiulong River tributaries in Zhangzhou, Fujian Province, China. The DNA GC content of the type strain is 40.4 mol%.

### Description of *Hydrocurvibacter sulfurireducens* sp. nov.

#### *Hydrocurvibacter sulfurireducens* (sul·fu·ri·re·du·cens. L. n. *sulfur*; L. part. adj. *reducens*, leading back, reducing; N.L. part. adj. *sulfurireducens*, reducing sulfur)

Cells are gram negative, rod shaped, and motile with a polar flagellum. Growth occurs at 15°C–40°C (optimum 32°C), pH 5.0–9.0 (optimum pH 6.5–7.0), and 2.0%–4.0% (wt/vol) NaCl (optimum 2.5%). Obligate chemolithoautotrophic growth occurs with H_2_, S^0^, thiosulfate, and sulfide as electron donors and S^0^, oxygen, or nitrate as an electron acceptor. Ammonium, nitrate, and atmospheric nitrogen are utilized as nitrogen sources. Organic substrates are not utilized as carbon sources and energy sources. Predominant fatty acids are C_16:1_ *ω*7*c*, C_18:1_ *ω*7*c*, and C_16:0_.

The type strain HSL-1716^T^ (=MCCC 1A17957^T^ = KCTC 25165^T^) was isolated from mangrove sediments of Jiulong River tributaries in Zhangzhou, Fujian Province, China. The DNA GC content of the type strain is 41.7 mol%.

## MATERIALS AND METHODS

### Sample collection, enrichment, and purification

The sediments were collected from the mangrove wetland of Jiulong River tributaries located in Zhangzhou (24°20′N, 117°45′E), Fujian Province, China. These samples are black and smell like rotten eggs, implying these sediments are enriched in sulfide. The samples were kept cool and returned to the laboratory. Each of 2 g (wet mass) was suspended with artificial seawater, and then 1 mL was transferred into 50 mL serum vials with 10 mL MMJHS medium under a gas phase mixture of 80% H_2_/18% CO_2_/2% O_2_ (200 kPa) and then incubated at 28°C according to the previous description (52). The serum vials were sealed with butyl rubber stoppers and aluminum crimp seals. After successful enrichment with MMJHS medium, cells were purified with the dilution-to-extinction technique. The purity of these cultures was confirmed by microscopic examination and 16S rRNA gene sequencing. MMJS medium consisted of NaCl (30 g/L), NH_4_Cl (0.25 g/L), KCl (0.33 g/L), CaCl_2·_2H_2_O (0.14 g/L), MgCl_2·_6H_2_O (4.18 g/L), K_2_HPO_4_ (0.14 g/L), NaHCO_3_ (1 g/L), Na_2_S_2_O_3·_5H_2_O (10 mM), Wolfe’s vitamins (1 mL/L), and trace element solution (10 mL/L) (12).

### Phenotypic and chemotaxonomic analysis

The cell morphology of the strains was observed with transmission electron microscopy (Model JEM-1230; JEOL, Japan). The physiological characterization of the isolate was detected on MMJHS medium (53). Unless otherwise stated, the experiments were conducted in triplicate. Growth temperature range was determined in MMJHS by incubating at 4°C, 10°C, 15°C, 20°C, 25°C, 28°C, 30°C, 33°C, 35°C, 37°C, 45°C, 50°C, and 60°C. The growth salinity range was examined by adjusting the concentrations of NaCl between 0% and 9% (wt/vol) at 0.5 (wt/vol) intervals. To determine the effect of pH on growth, the pH of MMJHS medium was adjusted from 4.5 to 9.0 with a 0.5 pH unit interval by different buffers. The effect of O_2_ on growth was examined by adjusting the oxygen concentration (0%, 1%, 2%, 4%, 6%, 8%, and 10% at 200 kPa and 20% at 100 kPa) in the headspace gas. In case of oxygen absence, 10 mM nitrate was added as a potential electron acceptor.

The ability for sulfur oxidation was tested in the MMJS medium using various sulfur compounds at different concentrations as the sole energy source, including thiosulfate (10 mM), sulfite (5 mM), thiocyanate (5 mM), tetrathionate (5 mM), elemental sulfur (1% wt/vol), or sulfide (50, 100, 500 µM and 1 mM) under N_2_/CO_2_. Molecular hydrogen was examined in MMJH medium in the absence of thiosulfate under a gas phase of 80% H_2_/20% CO_2_. The ability for sulfur disproportionation was tested in MMJS medium supplemented with 10 mM thiosulfate or 5 g/L elemental sulfur in the presence of 20 mM ferrihydrite as a sulfide-scavenging agent. Growth by disproportionation was possible only in the presence of ferrihydrite [poorly crystalline Fe(III) oxide], which bound sulfide, forming a reduced black non-magnetic precipitate, probably Fe(II) sulfide (19, 54). To determine the utilization of other electron acceptors, thiosulfate (10 mM), tetrathionate (10 mM), sulfite (2 mM and 10 mM), elemental sulfur (1%, wt/vol), nitrate (10 mM), nitrite (1 and 5 mM), ferric citrate (20 mM), selenate (5 mM), and arsenate (5 mM) were tested with MMJHS medium under 80% H_2_/20% CO_2_. Heterotrophic growth was examined in MMJHS medium without NaHCO_3_ under 80% N_2_/20% CO_2_. Each of the following potential organic carbon sources was tested: 0.1% (wt/vol) peptone, yeast extract, tryptone, starch, casein, and casamino acids; 5 mM formate, acetate, propionate, citrate, tartrate, fumarate, succinate, malate, and pyruvate; 5 mM of each of 20 amino acids; and 0.02% (wt/vol) glucose, galactose, sucrose, fructose, lactose, maltose, and trehalose. In an attempt to determine the alternative energy source, these organic compounds were used as an energy source in MMJ medium to replace thiosulfate under 80% N_2_/20% CO_2_.

For analyses of fatty acids, cells grown on medium were saponified, methylated, and extracted using the standard MIDI (Sherlock Microbial Identification System, v.6.0B) protocol (52). The fatty acids were analyzed by gas chromatography (Agilent Technologies 6850) and identified using the TSBA6.0 database of the Microbial Identification System.

### Phylogenetic analysis and genome sequencing

DNA isolated from different strains was extracted using the method described by Jiang et al. (52), and the 16S rRNA gene was amplified by PCR using primers Bac27F and 1492R (52). The similarity of 16S rRNA gene sequences was determined using the EzBioCloud server and National Center for Biotechnology Information (NCBI) database, and sequences of reference strains were downloaded for the phylogenetic analysis. Sequence comparison was performed using CLUSTAL_W, and phylogenetic trees were constructed using MEGA v.6.0 according to the maximum likelihood method (55). The complete genome of seven strains was sequenced by Tianjin Biochip Corporation (Tianjin, China), using the single-molecule real-time technology on the Pacific Biosciences platform. The sequenced reads were filtered, and high-quality paired-end reads were assembled to construct a circular genome with SOAPdenovo v.2.04 (56). The GC content was determined according to the genome sequence. Gene prediction was carried out by the Glimmer program (57). Gene annotation was performed by the Rapid Annotation using Subsystem Technology server and NCBI Prokaryotic Genomes Annotation Pipeline. Genomic relatedness was evaluated by different algorithms for genome-to-genome comparison. The ANI value between two genomes was calculated by the web service of EZGenome (16). The predicted *in silico* DNA-DNA hybridization values were determined online using the Genome-to-Genome Distance Calculator (16). The phylogenomic tree was constructed based on an up-to-date 92 bacterial core gene sets by UBCG v.3.0 (15). The whole-genome sequences of reference taxa were obtained from the NCBI database. The 92 concatenated gene sequences were extracted, aligned, and concatenated within UBCG using default parameters. A maximum-likelihood phylogenetic tree was inferred using RAxML v.8.2.11 with the GTR + CAT model (58). Furthermore, taxonomic annotations of each strain were performed using GTDB-Tk v.2.4.0 with the “classify_wf” workflow (default parameters) against the reference database (R220) (59). The completeness and contamination of the genomes from isolated strains were estimated using CheckM v.1.0.18 (60).

### 16S rRNA gene amplicon sequencing

Genomic DNA was extracted from 0 to 20 cm mangrove sediments at 2 cm intervals using the DNeasy PowerMax Soil Kit (12988-10; QIAGEN, Germany) according to the manufacturer’s instructions (33). The V3–V4 region of the bacterial 16S rRNA gene was amplified with the universal primers 338F and 806R (38). Amplicon sequencing was performed on a MiSeq platform (Illumina) using 2 × 300 bp chemistry. The amplicon sequence variant (ASV) was obtained after denoising and removal of chimeras by the DADA2 algorithm (61), and classified using a naive Bayesian classifier in QIIME2 (feature-classifier classifysklearn) with a confidence score of 0.7 (-p-confidence) against the SILVA v138 database (62).

### Metatranscriptomic analysis

Total RNA was extracted from 0 to 20 cm mangrove sediments at 2 cm intervals using the RNeasy PowerSoil Total RNA Kit (12866-25, QIAGEN) according to the manufacturer’s protocol. RNA concentration and quality were evaluated using a Qubit 2.0 Fluorometer (Life Technologies, USA) and gel electrophoresis, respectively. Whole transcriptome amplification of total RNA was carried out using the RNA REPLI-g Cell WGA & WTA Kit (150054c, QIAGEN) according to the manufacturer’s protocol. To enrich messenger RNA (mRNA), ribosomal RNA was depleted from total RNA using the RiboCop rRNA Depletion Kit (Lexogen, Austria) (38). Whole mRNA-Seq libraries were generated by Majorbio Biotechnology Co. Ltd., (Shanghai, China) using the NEBNext Ultra Nondirectional RNA Library Prep Kit (E6111; New England Biolabs, USA) following the manufacturer’s instructions. The constructed libraries were sequenced on a NovaSeq 6000 platform (Illumina), and 150 bp paired-end reads were generated. Raw metatranscriptomic reads were quality filtered using fastp v.0.23.2 with default parameters (63). The reads corresponding to ribosomal RNAs were removed using SortMeRNA v.4.3.4 (64) with default parameters with the smr_v4.3_default_db database. Then, these high-quality metatranscriptomic reads from 10 sediment samples were mapped to function genes involved in carbon, nitrogen, sulfur, hydrogen, and oxygen metabolisms from the seven strains using Salmon v.1.9.0 (65) in mapping-based mode (parameters: -validate Mappings -meta). The expression level of each gene was normalized to TPM. TPM allowed comparisons of gene expression from sample to sample by normalizing different sequencing depths.

### Global distribution pattern

To reveal the global distribution of all seven strains in this study, we collected microbial data containing all seven strains reads by using the IMNGS (https://www.imngs.org/) database (50), which pigeonholes all available raw sequence read archives retrieved from the International Nucleotide Sequence Database (GenBank, DDBJ, and EMBL) and allows users to conduct comprehensive searches of small-subunit rRNA gene sequences. In this study, representative sequences (the nearly full-length 16S rRNA gene) were subjected to a similarity search against the IMNGS database using a similarity threshold of 97% and a minimum length of 200 bp (66). The samples were retrieved, and their metadata, including source, latitude, longitude, and depth of seawater, were obtained from NCBI (Table S10).

## Data Availability

The following are available at the National Center for Biotechnology Information: all metatranscriptomic raw reads used in this study (accession nos. SAMN37429165-37429174), the 16S rRNA gene sequences from seven isolated strains (accession nos. OQ102620, OQ102624-OQ102628, and OL454755), and the genome sequences of seven strains (accession nos. CP147914-CP147919 and CP087998).

## SUPPLEMENTAL MATERIAL

Fig S1 spectrum.00367-25-s0001.pdf Transmission electron micrographs of the cells of strains HSL1-2 (A), HSL-1656 (B), HSL-3221 (C), HSL1-6 (D), HSL3-7 (E), HSL3-2 (F) and HSL-1716 (G).

TABLE S1 spectrum.00367-25-s0002.xlsx The 16S rRNA gene identity between the seven strains and fifteen closely related type strains within the *Sulfurimonas* genus.

ASM does not own the copyrights to Supplemental Material that may be linked to, or accessed through, an article. The authors have granted ASM a non-exclusive, world-wide license to publish the Supplemental Material files. Please contact the corresponding author directly for reuse.
