# Supplementary material for: Cultivation and metabolic versatility of novel and ubiquitous chemolithoautotrophic Campylobacteria from mangrove sediments
Source: Microbiol Spectr. 2025 Jul 23;13(9):e00367-25. doi: 10.1128/spectrum.00367-25 (PMC12403661; doi:10.1128/spectrum.00367-25)

Cultivation and metabolic versatility of novel and ubiquitous chemolithoautotrophic *Campylobacteria* from mangrove sediments

Liang Cui[1#], Yangsheng Zhong[1#], Yufei Li[1], Stefan M. Sievert[2], Zhaobin Huang[3], Maxim Rubin-Blum[4], Xiaxing Cao[1], Yong Wang[1], Zongze Shao[1,5], Qiliang Lai[1], Shasha Wang[1*] and Lijing Jiang[1*]

[1] Key Laboratory of Marine Genetic Resources, Third Institute of Oceanography, Ministry of Natural Resources of PR China, Xiamen 361005, China

[2] Biology Department, Woods Hole Oceanographic Institution, Woods Hole, MA 02543, USA

[3] College of Oceanology and Food Science, Quanzhou Normal University, Quanzhou 362000, China

[4] Biology Department, National Institute of Oceanography, Israel Oceanographic and Limnological Research (IOLR), Haifa 3108000, Israel

[5] Fujian Ocean Innovation Center, Xiamen 361102, China

*Correspondence: Shasha Wang (wangshasha@tio.org.cn)

Lijing Jiang (Jianglijing@tio.org.cn)

[#] Authors contributed equally to this manuscript.

Fig. S1. Transmission electron micrographs of the cells of strains HSL1-2 (A), HSL-1656 (B), HSL-3221 (C), HSL1-6 (D), HSL3-7 (E), HSL3-2 (F) and HSL-1716 (G).

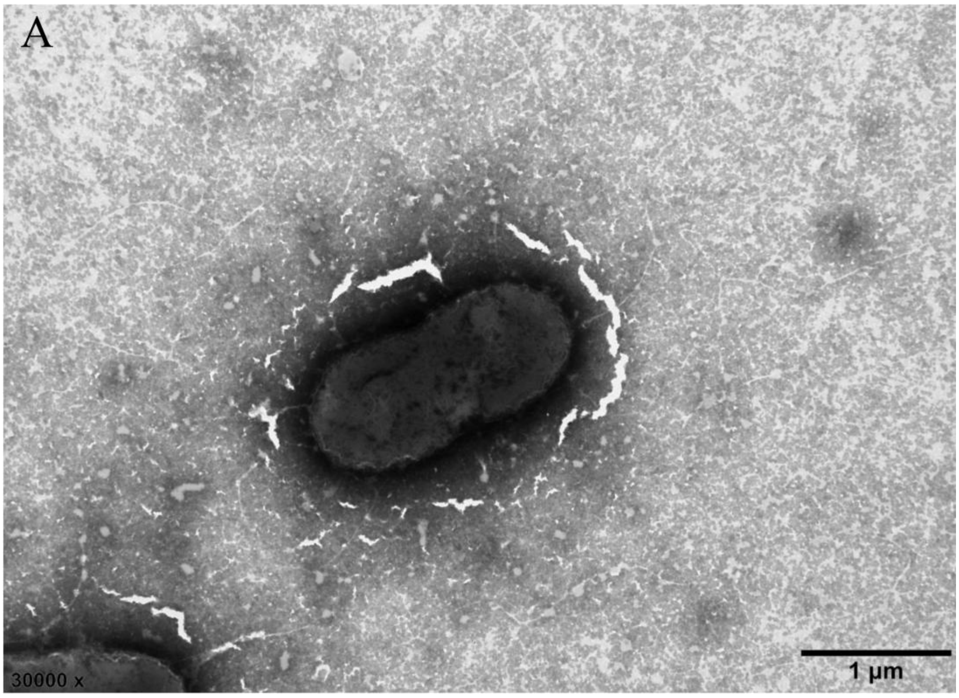

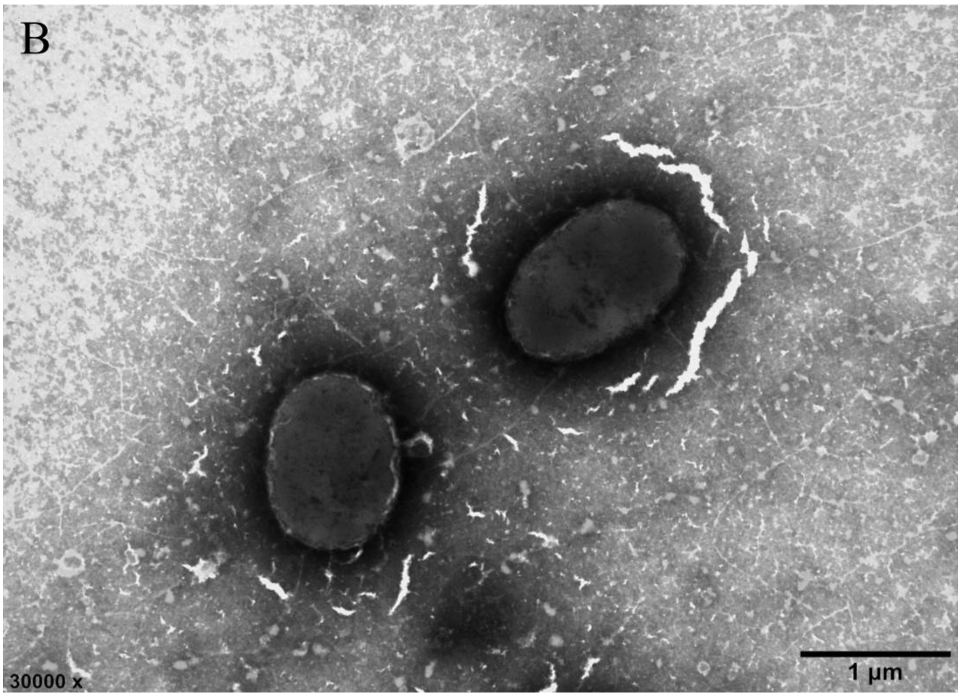

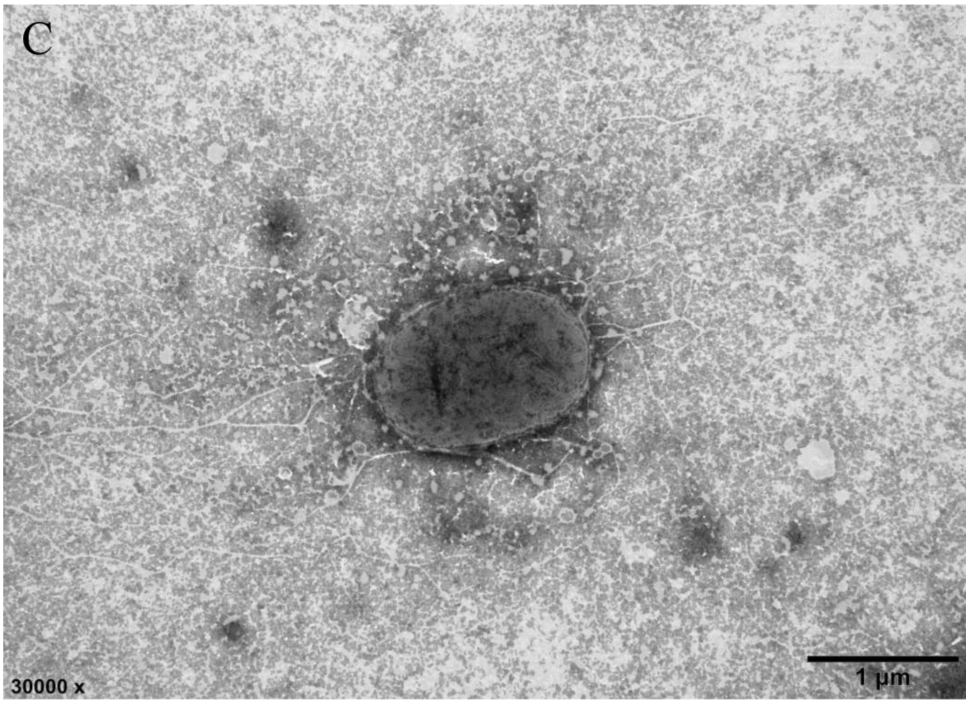
C
30000 x
1 µm

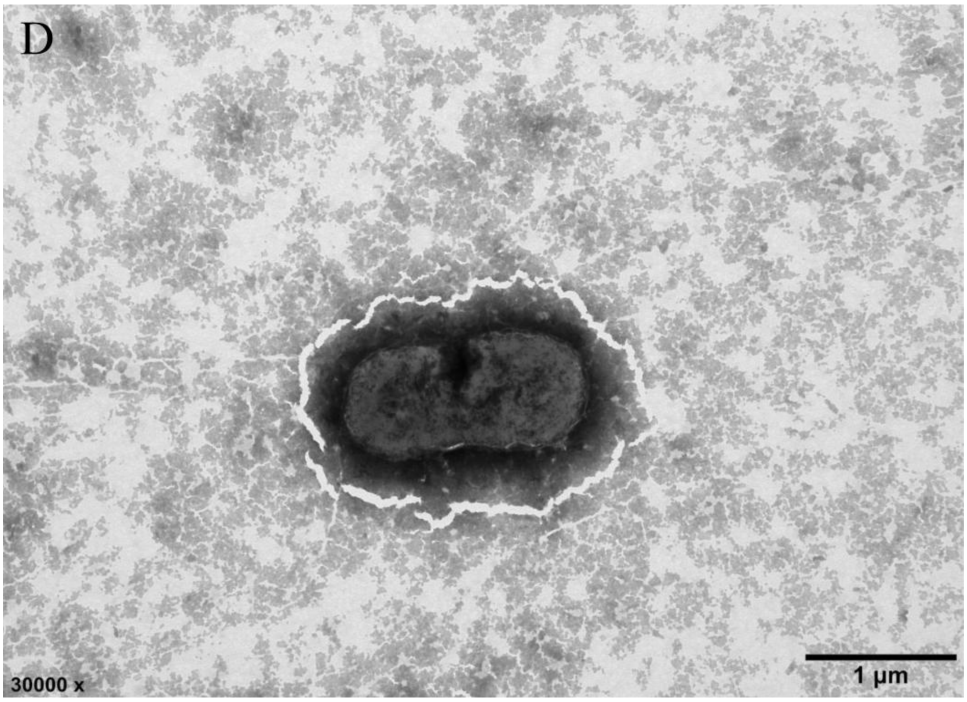
D
30000 x
1 µm

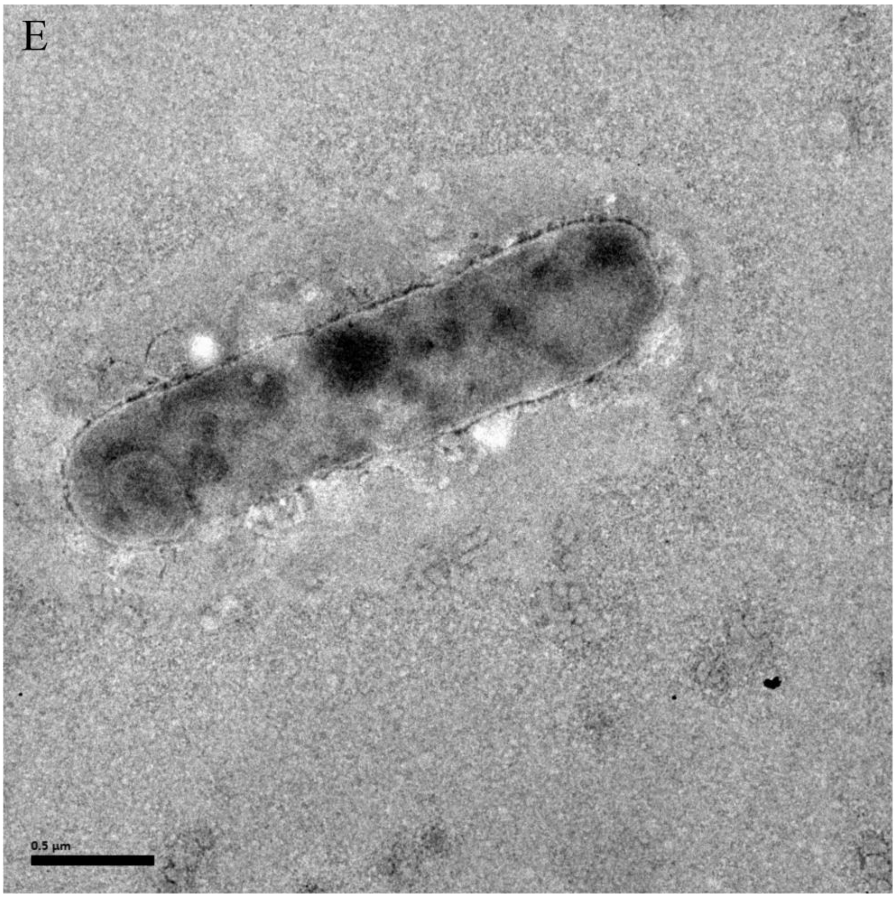
E
0.5 μm

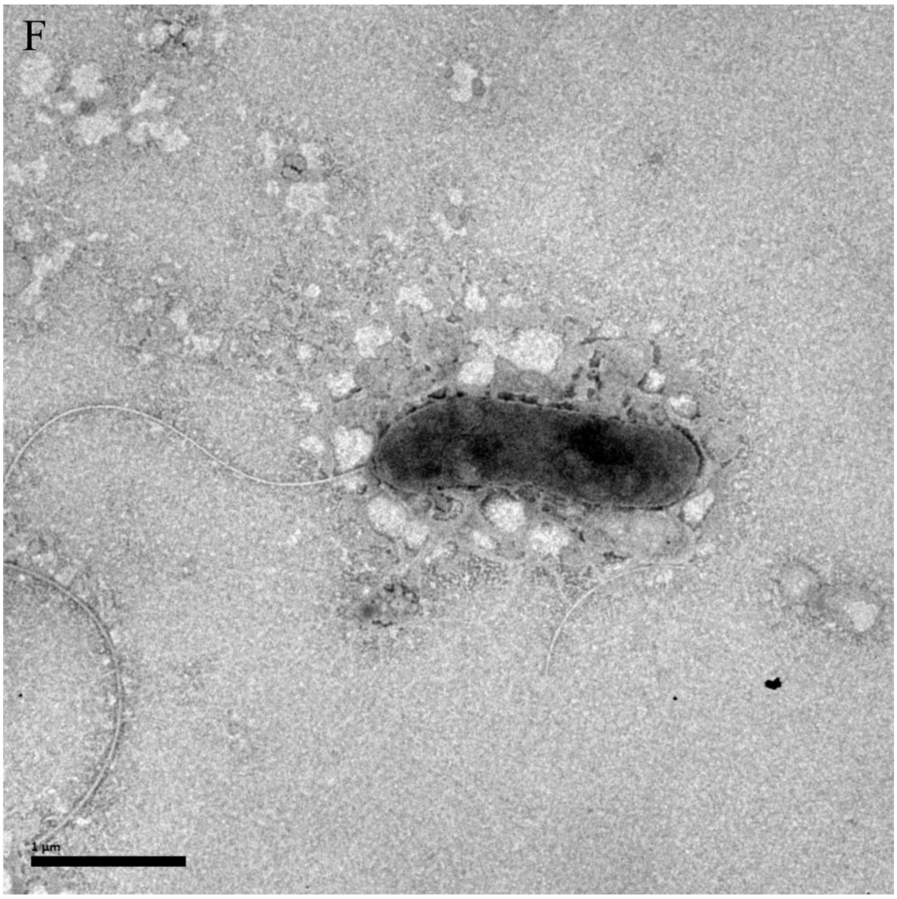
F
1 μm

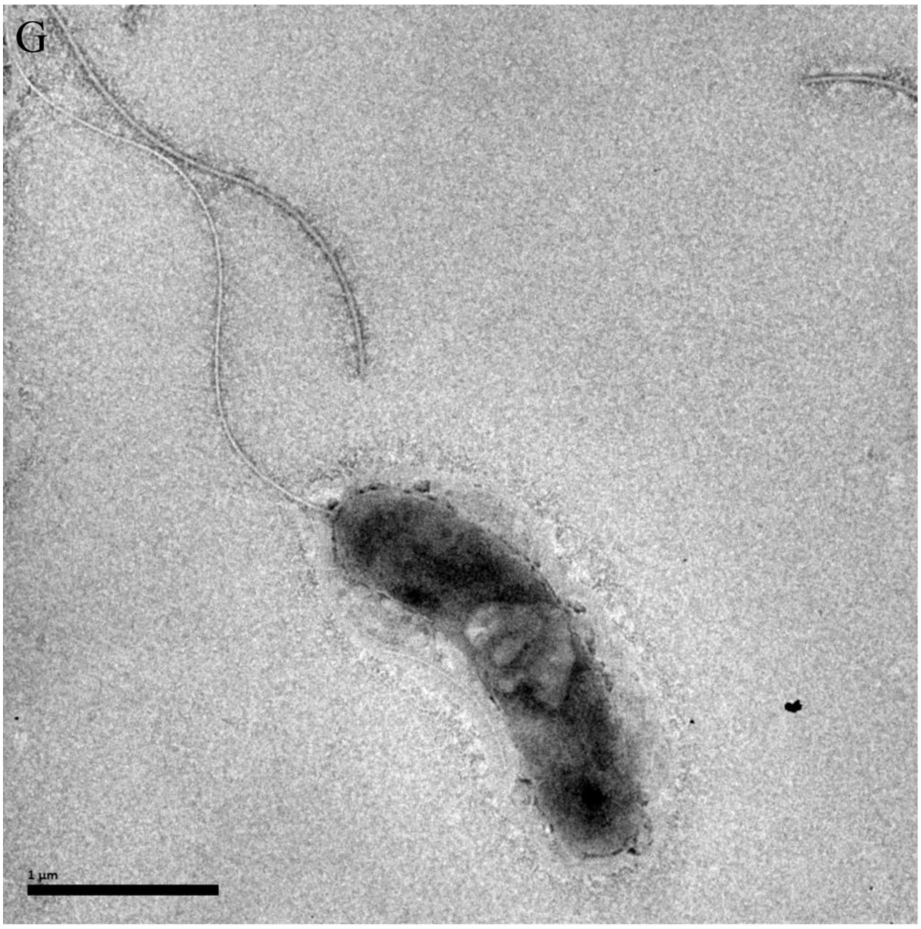
G
1 µm

Fig. S2 The occurrence of sulfate reduction coupled with hydrogen oxidation in *S. jiaomeiensis* HSL3-7^T. (A) The visual changes of the medium before and after culture with the appearance of black sulfide. (B) The cell growth and concentrations of sulfate and sulfide during the incubation. Error bars represent the range of measurements from duplicate cultures.

A

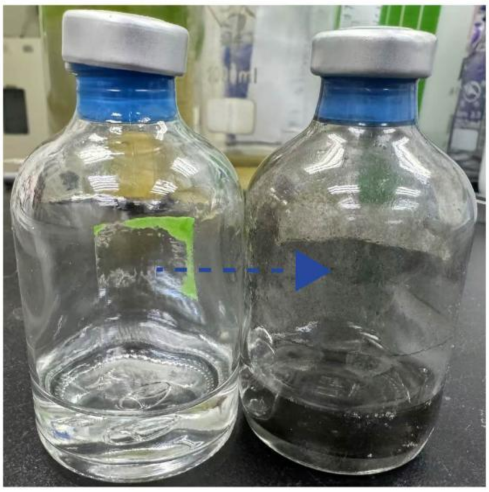

B

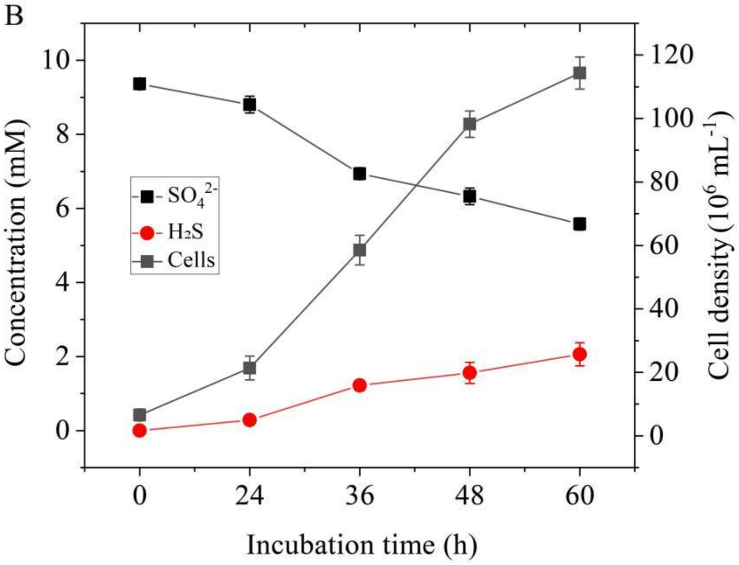

Supplement: Fig S1 — Transmission electron micrographs of the cells of strains HSL1-2 (A), HSL-1656 (B), HSL-3221 (C), HSL1-6 (D), HSL3-7 (E), HSL3-2 (F) and HSL-1716 (G). [file spectrum.00367-25-s0001.pdf]
